# Changes in frontal plane kinematics over 12-months in individuals with the Percutaneous Osseointegrated Prosthesis (POP)

**DOI:** 10.1371/journal.pone.0281339

**Published:** 2023-02-22

**Authors:** Benjamin J. Darter, E. Daniel Syrett, K. Bo Foreman, Erik Kubiak, Sarina Sinclair

**Affiliations:** 1 Department of Physical Therapy, Virginia Commonwealth University, Richmond, VA, United States of America; 2 Physical Medicine and Rehabilitation Service, Central Virginia Veterans Health Care System, Richmond, VA, United States of America; 3 Department of Physical Therapy, University of Utah, Salt Lake City, UT, United States of America; 4 Department of Orthopaedics, University of Utah, Salt Lake City, UT, United States of America; 5 Research Service, Salt Lake City Health Care System, Salt Lake City, UT, United States of America; 6 Department of Orthopedic Surgery, University of Nevada Las Vegas, Las Vegas, NV, United States of America; West Park Healthcare Centre, CANADA

## Abstract

**Background:**

A bone-anchored prosthesis (BAP) eliminates the need for a conventional socket by attaching a prosthesis directly to the user’s skeleton. Currently, limited research addresses changes in gait mechanics post BAP implantation.

**Objective:**

Examine changes in frontal plane movement patterns after BAP implantation.

**Methods:**

Participants were individuals with unilateral transfemoral amputation (TFA) enrolled in the US Food and Drug Administration (FDA) Early Feasibility Study examining the Percutaneous Osseointegrated Prosthesis (POP). The participants completed overground gait assessments using their conventional socket and at 6-weeks, 12-weeks, 6-months, and 12-months following POP implantation. Statistical parameter mapping techniques were used in examining changes in frontal plane kinematics over the 12-months and differences with reference values for individuals without limb loss.

**Results:**

Statistically significant deviations were found pre-implantation compared to reference values for hip and trunk angles during prosthetic limb stance phase, and for pelvis and trunk relative to the pelvis angles during prosthetic limb swing. At 6-weeks post-implantation, only the trunk angle demonstrated a statistically significant reduction in the percent of gait cycle with deviations relative to reference values. At 12-months post-implantation, results revealed frontal plane movements were no longer statistically different across the gait cycle for the trunk angle compared to reference values, and less of the gait cycle was statistically different compared to reference values for all other frontal plane patterns analyzed. No statistically significant within-participant differences were found for frontal plane movement patterns between pre-implantation and 6-weeks or 12-months post-implantation.

**Conclusions:**

Deviations from reference values displayed prior to device implantation were reduced or eliminated 12-months post-implantation in all frontal plane patterns analyzed, while within-participant changes over the 12-month period did not reach statistical significance. Overall, the results suggest the transition to a BAP aided in normalizing gait patterns in a sample of relatively high functioning individuals with TFA.

## Introduction

Osseointegration for individuals with transfemoral amputation (TFA) has received significant attention in recent years. This surgical procedure consists of implanting a bone-anchored prosthesis (BAP) in the residual femur that extends through the skin surface to link the bone directly to other components of the prosthetic limb [1, 2]. Conventional socket and suspension systems create common problems such as residual limb pain, skin injury, sitting discomfort and limb-socket instability [3, 4], and bypassing the socket using a direct connection may reduce these issues.

Research involving BAPs in individuals with TFA often focused on quantifying adverse events with the implanted device (i.e., loosening, infection, peri-prosthetic fracture, device breakage), force loaded on the implant during activity, and the impact of a BAP on outcomes such as hip range of motion, sitting comfort, and quality of life [5–12]. Overall, the literature suggests that a BAP has a positive impact on functional and clinical outcomes for individuals with TFA, as the transition from a conventional socket to a BAP was associated with increased activity levels, increased prosthesis use, improved mobility, reduced energy expenditure, and improvement in functional gait measures [5, 6, 13].

Fewer studies report the effect of a BAP on gait biomechanics. Frossard et al. and Pinard et al. found the cadence, gait cycle duration, and sound side step lengths in those with a BAP were equivalent or improved compared to those with conventional socket and suspension systems [14, 15]. Elsewhere, Tranberg et al. examined sagittal plane gait kinematics in individuals 2 years post-BAP implantation and found improved prosthetic side hip extension and reduced pelvic anterior tilt compared to pre-BAP implantation [16]. While these studies suggest a BAP was beneficial, the analyses were limited to temporospatial measures and sagittal plane movements. Kinematic deviations are also common in other planes of movement among those with TFA. Previous work shows frontal plane deviations include a widened step width, abducted hip position, and increased lateral trunk lean during prosthetic side stance [17, 18]. These deviations are likely related to problems stemming from socket use [3], and a transition from a conventional socket device to a BAP may therefore also reduce frontal plane gait deviations. Reductions in these deviations may serve to improve gait function and efficiency, and thus an investigation of the effect of a BAP on frontal plane movements is warranted. However, no study to date has reported frontal plane gait characteristics in those with TFA before and after implantation with a BAP.

The purpose of this study was to compare frontal plane gait kinematics of the trunk, pelvis, and hip pre-, 6-weeks post-, and 1-year post-implantation with a BAP in individuals with TFA. We hypothesized that a transition from a conventional socket and suspension system to a BAP would facilitate frontal plane gait kinematics more closely resembling individuals without an amputation.

## Materials and methods

### Participants

Study participants were individuals with traumatic unilateral TFA enrolled in a United States Food and Drug Administration (FDA) Early Feasibility Study (EFS) for a new BAP device named the Percutaneous Osseointegrated Prosthesis (POP) [19, 20]. This Level II therapeutic prospective, single-center, nonrandomized clinical trial (ClinicalTrials.gov Identifier: NCT02720159) was approved to enroll a total of 10 individuals. A nationwide sample of Veterans receiving care within the Veterans Health Administration system were identified and recruited to the clinical trial between December 2015 and May 2017. These individuals were 18 years of age or older and had a non-dysvascular disease related TFA at least 6 months prior. A complete list of clinical trial inclusion and exclusion criteria was reported in a separate manuscript describing the overall outcomes of the FDA EFS [19]. Participants provided written informed consent prior to completing the testing procedures approved by the University of Utah Institutional Review Board.

### Data collection

Testing was completed on an overground walkway. Each participant was instructed to walk at a comfortable self-selected walking speed (SSWS). Full-body kinematic data were collected for walking at 200 Hz using a six-degrees of freedom marker set [21] and a 10-camera motion capture system (Vicon, Oxford, UK). Analog data from 2 AMTI OR6-7 force plates (Advanced Medical Technologies Inc., Watertown, MA) embedded in the walkway were synchronously collected at 1000 Hz. A sufficient number of trials were completed in order to obtain a minimum of 7 complete stance cycles on a force plate for each limb (range 7–19 trials per limb). During testing the participants were permitted to use an assistive device (e.g., cane or walker) as needed. Testing was completed using their customary prosthesis with a conventional socket, and then at 6-weeks, 12-weeks, 6-months, and 12-months after the POP implant surgery.

### Data reduction

Data from the 71 markers and 2 force plates were lowpass-filtered in Visual 3D motion analysis software (C-Motion, Germantown, MD) using a 4^th^ order zero-lag Butterworth filter with cutoffs (6Hz and 25Hz, respectively) determined by visual inspection. A 15-segment full-body model was created and the frontal plane kinematics of the hip joint relative to the pelvis segment (hip adduction angle), pelvis segment relative to the lab coordinate system (pelvis-lab angle), trunk segment relative to the lab (trunk-lab angle), and trunk segment relative to the pelvis segment (trunk-pelvis angle) were extracted from the walking trials. Force data were used to identify initial contact and toe-off gait events with gait cycles defined as consecutive initial contacts of the same limb. Kinematic data were temporally normalized to the gait cycle and averaged. Step width was calculated as the distance along the mediolateral plane between the proximal ends of each foot segment for consecutive heel strikes. Gait speed was calculated by dividing the stride length by the stride time.

### Statistical analysis

The time points of interest were the pre-implantation test, the 6-week post-implantation test, and the 12-month post-implantation test. Group data from other time points are presented for descriptive purposes but were not included in statistical testing due to the small sample size. A one-way ANOVA was used to assess the effect of testing period on step width and a paired t-test was used to evaluate changes in walking speed between pre-implantation and the 12-month post-implantation visit. Both tests were conducted using JMP v16 (SAS Institute, Cary, NC). Frontal plane kinematics were statistically analyzed with one dimensional Statistical Parametric Mapping (SPM) techniques. This approach allows for analysis of the entire gait cycle curve by calculating a test statistic at each gait cycle percentage and utilizing random field theory to assess significance [22]. This method prevents reduction of the data to zero-dimensional values such as maxima or ranges, thus diminishing both selection bias and false positives [22, 23]. SPM tests were conducted utilizing the “spm1d” open-source package [24] in MATLAB r2021a (MathWorks, Inc., Natick, Massachusetts, USA). Two different set of comparisons were made using the SPM technique: 1) Unpaired two-sample, two-tailed SPM t-tests were used to compare the pre-implantation, 6-week post-implantation, and the 12-month post-implantation values to reference data for individuals without TFA. The reference dataset was derived from previously collected data of 20 individuals without limb amputation walking at 1.5 m/s on a treadmill [25]; 2) Paired, two-tailed SPM t-tests were used to compare prosthetic side frontal plane angles between pre-implantation and 6-week post-implantation as well as between pre-implantation and 12-month post-implantation visits. For all tests, normality was verified using a D’Agostino-Pearson K^2^ test of the residuals. If the normality assumption was violated, then a non-parametric permutation SPM test was used instead [26]. Testing was evaluated at the significance level of 0.10 based on recommendations for setting the significance level for exploratory or preliminary studies [27]. Results are presented as group means, but figures with individual participant pre-implantation, 6-weeks post-implantation, and 12-months post-implantation data are included as supporting information (S1 File).

## Results

### Participants

**Fig 1:**
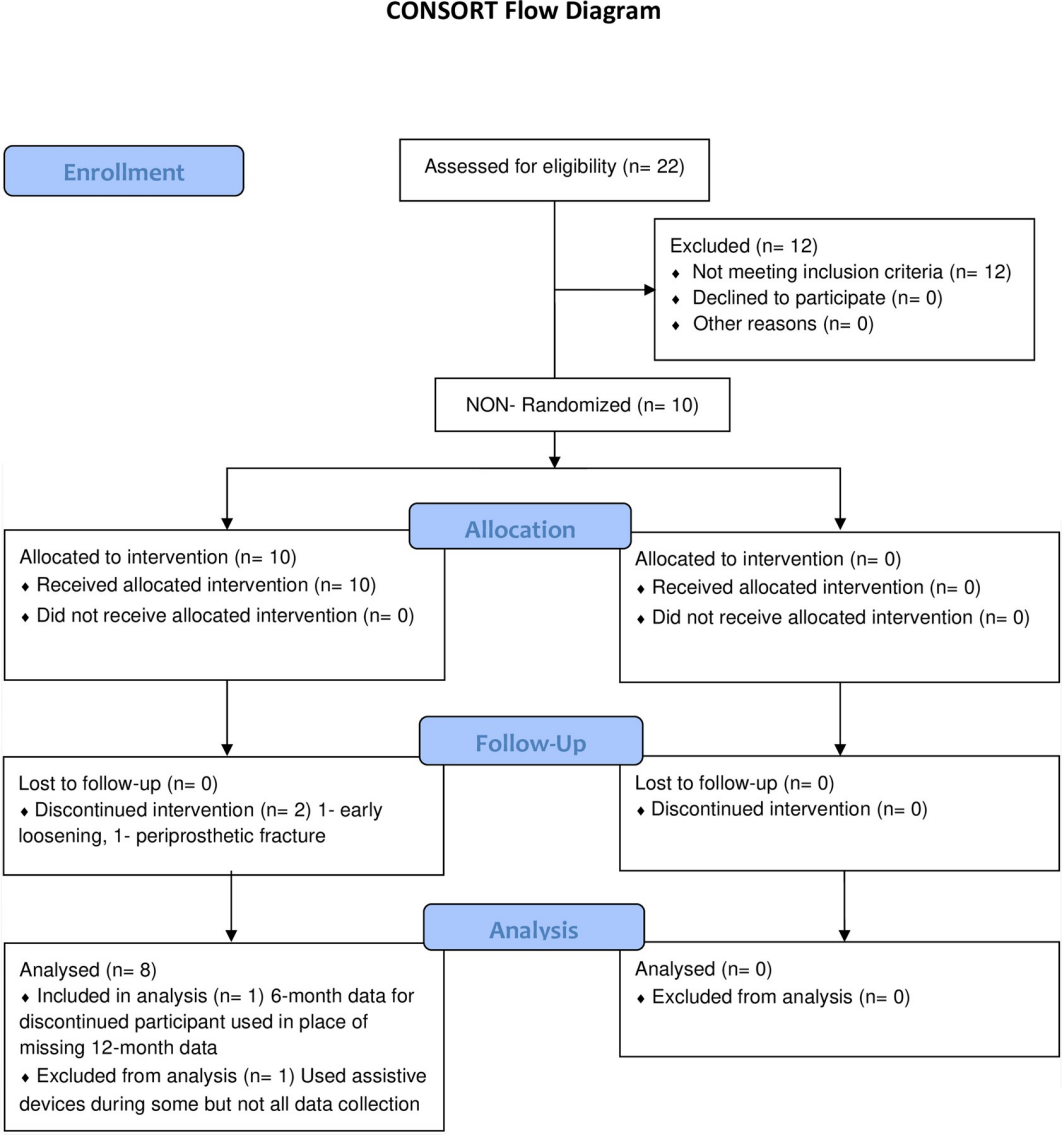
CONSORT flow diagram.

Ten Caucasian males with traumatic unilateral TFA were enrolled in the FDA EFS for the POP device. Eight individuals completed all testing sessions. Two participants experienced complications with the implant and withdrew prior to the 12-month testing session. 6-month post-implantation visit data for one of the withdrawn participants were used in the statistical analyses in place of 12-month data. Additionally, one participant used assistive devices during the initial and 6-week post-implantation data collections but no assistive device at the other collections. This participant’s data were excluded due to the potential effect of the assistive devices on gait mechanics. As a result, statistical analyses were conducted with pre-implantation and 12-month post-implantation data from 7 participants as well as pre-implantation and 6-month post-implantation data from 1 participant. Full demographic information for the 8 participants can be found in Table 1, and the CONSORT flow diagram can be found in Fig 1. Pre-implantation, the group walked with an average SSWV of 1.23 m/s. At the 12-month pos-implantation visit, the average SSWV was 1.36 m/s, but this increase was not statistically significant compared to pre-implantation (p = 0.33).

**Table 1:** Data for Participants at their pre-POP implantation session.

	Sex	Age (yrs)	Height (m)	Weight (kg)	Time Since Amputation (years)	Self-Selected Walking Speed (m/s)	Prosthetic Knee	Prosthetic Foot	Medicare K-Level
**1**	Male	44	1.83	73.9	13	1.46	Ossur Rheo	Ossur Pro-Flex Pivot	4
**2**	Male	35	1.80	96.6	3	1.26	Ottobock Genium	Ottobock Triton	4
**3**	Male	49	1.73	78.5	13	1.26	Ottobock C-Leg	Ottobock Triton Low Profile	3
**4**	Male	61	1.78	83.8	6	1.19	Ottobock C-Leg	Ottobock Axtion	3
**5**	Male	41	1.85	104.5	13	1.65	Ottobock X3	Fillauer Wave Sport	4
**6**	Male	46	1.76	103.7	11	1.39	Ottobock X3	Freedom Innovations Renegade XL	4
**7**	Male	32	1.71	63.4	9	1.02	Ottobock X3	Ability Dynamics Rush Renegade	3
**8**	Male	64	1.72	96.9	17	1.29	Ottobock X3	Ability Dynamics Rush Low Profile	4
**Mean**		46.5	1.77	87.7	10.6	1.32			
**SD**		11.5	0.05	15.0	4.5	0.19			

SD: Standard Deviation

### Frontal plane spatial results

The group demonstrated a mean step width of 0.21 (SD: ±0.06) m at the pre-implantation tests. The step width decreased to 0.19 (SD: ±0.04) m at the 6-week implantation visit and to 0.18 (SD: ±0.05) m by the 12-month post-implantation tests. The effect of time period on step width did not reach statistical significance (p = 0.53).

### Frontal plane kinematic results

#### Hip adduction angle

**Fig 2:**
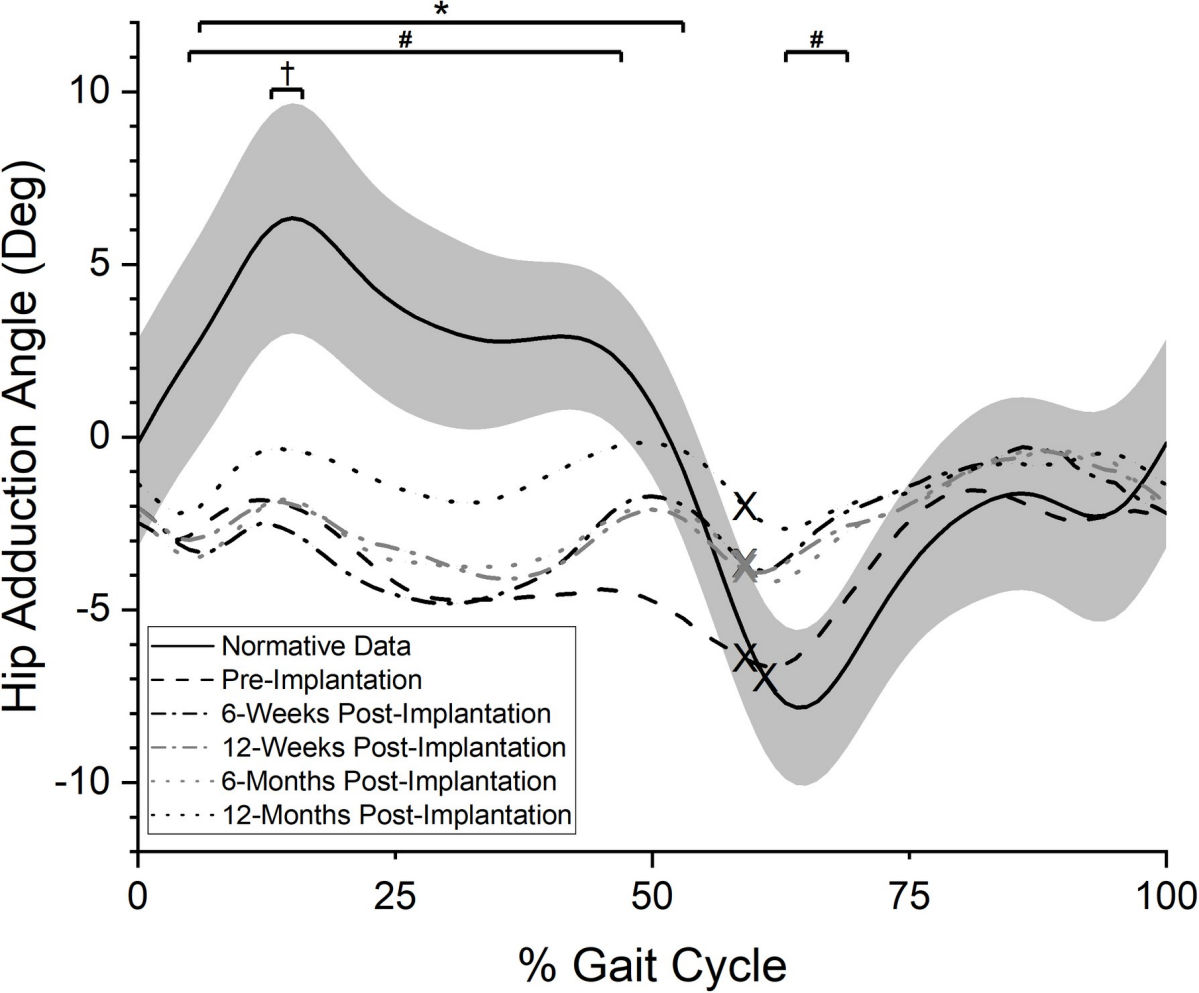
Frontal plane hip angle. Averaged, normalized frontal plane hip angle values over the prosthetic side gait cycle (0% = POP side heel strike, 100% = subsequent POP side heel strike). “X” marks represent the mean toe-off event for each curve. Positive values indicate hip adduction relative to the pelvis, while negative values indicate hip abduction relative to the pelvis. Solid black line indicates reference values for individuals without TFA while grey band indicates plus or minus one standard deviation of the reference data. *: period of statistically significant difference in pre-implantation test values from reference data (p<0.01); #: periods of statistically significant difference in 6-week post-implantation test values from reference data (p<0.01 and p = 0.08); †: period of statistically significant difference in 12-month post-implantation test values from reference data (p = 0.04).

Descriptively, participants demonstrated prosthetic side hip abduction throughout the stance phase with a transition to a minimally adducted position during swing at the pre-implantation tests (Fig 2). Large deviations from reference values were observed during stance with a statistically significant difference in hip angle during mid-stance through pre-swing (6–53% of the gait cycle, p<0.01). The hip angle during swing at the pre-implantation tests was within the reference values’ confidence interval and was not statistically different. At the 6-week post-implantation visit, there was a statistically significant difference in hip angle from reference values during stance (5–47% of the gait cycle, p<0.01) and for a brief period during early swing (63–69% of the gait cycle, p = 0.08). The comparison between the 6-week post-implantation frontal plane hip data and pre-implantation values did not reach statistical significance (p>0.10). The overall post-implantation hip movement pattern was qualitatively maintained until the 12-month session in which the hip reached a more adducted position during stance and approached the reference data curve. However, the hip adduction angle was still statistically different than the reference data curve for a small period during early midstance (13–16% of the gait cycle, p = 0.04). Statistical testing revealed no significant differences across the gait cycle for comparisons of the frontal plane prosthetic side hip angles pre- and 12-month post-implantation (p>0.10).

#### Pelvis-lab angle

**Fig 3:**
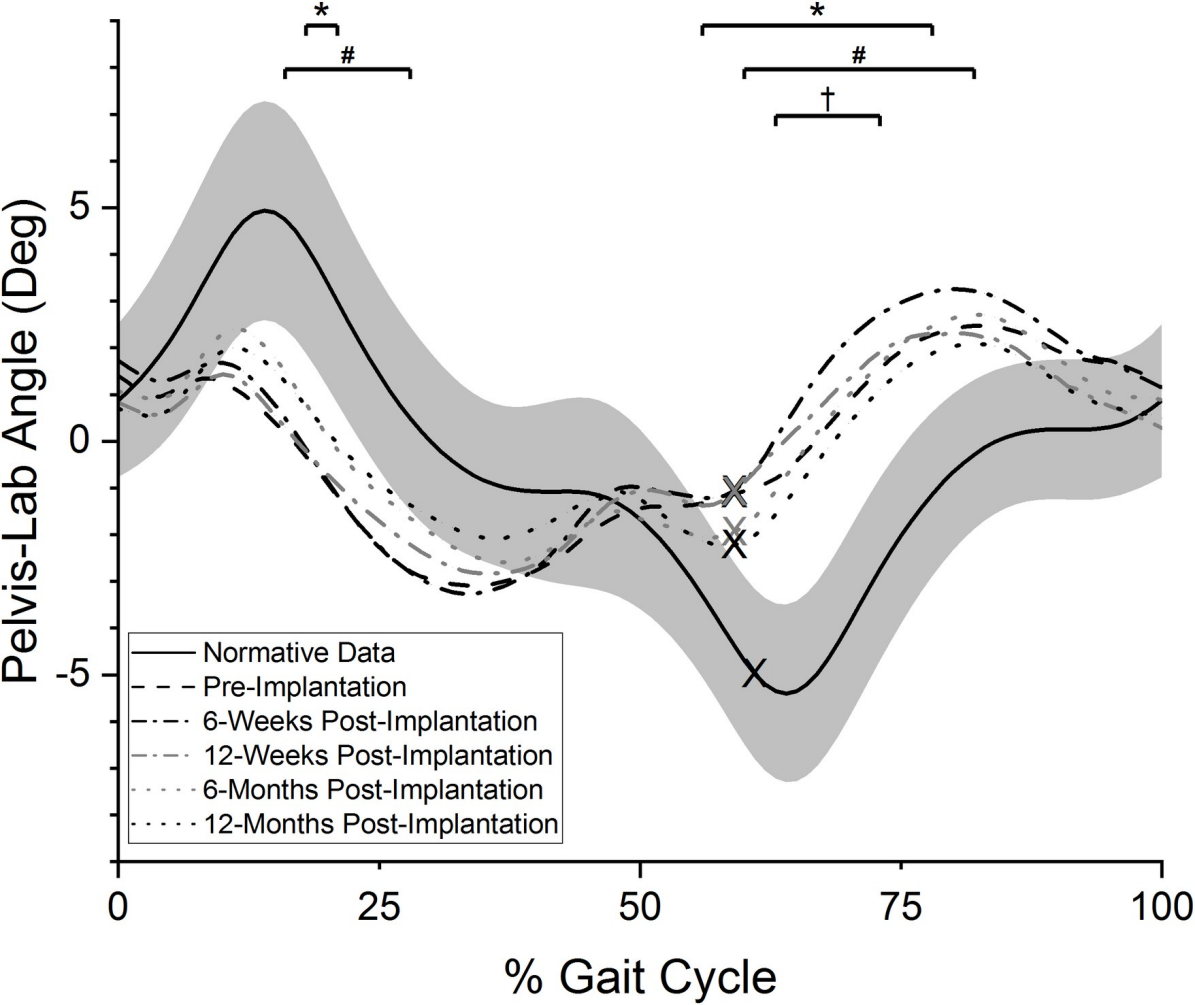
Frontal plane pelvis angle. Averaged, normalized frontal plane pelvis angle values over the prosthetic side gait cycle (0% = POP side heel strike, 100% = subsequent POP side heel strike). “X” marks represent the mean toe-off event for each curve. Positive values indicate ipsilateral pelvic hike relative to the lab space, while negative values indicate ipsilateral pelvic drop relative to lab space. Solid black line indicates reference values for individuals without TFA while grey band indicates plus or minus one standard deviation of the reference data. *: periods of statistically significant difference in pre-implantation test values from reference data (p = 0.05 and p = 0.01); #: periods of statistically significant difference in 6-week post-implantation test values from reference data (p = 0.05 and p = 0.01); †: period of statistically significant difference in 12-month post-implantation test values from reference data (p = 0.06).

Pre-implantation, a contralateral hike of the pelvis was developed early in stance on the prosthetic limb and was retained throughout the remainder of the stance phase (Fig 3). This was a statistically significant difference from reference values during 18–21% of the gait cycle (p = 0.05). Conversely, the pelvis demonstrated an ipsilateral hike during prosthetic limb swing. This pattern was statistically different from reference values during late pre-swing to mid-swing (56–78% of the gait cycle, p = 0.01). At the 6-week post-implantation visit, statistically significant deviations from reference values during both the stance (16–28% of the gait cycle, p = 0.05) and the swing phases remained (60–82% of the gait cycle, p = 0.01). However, this pattern was not statistically different than pre-implantation values (p>0.10). A similar movement pattern was observed each subsequent visit, though a progressive small reduction in the contralateral hike was observed. At the 12-month post-implantation test, a reduced percentage of the gait cycle statistically differed between the study participants and the reference data (63–73% of the gait cycle, p = 0.06). There was no statistically significant difference between 12-months post-implantation and pre-implantation frontal plane pelvis movement patterns (p>0.10).

#### Trunk-lab angle

**Fig 4:**
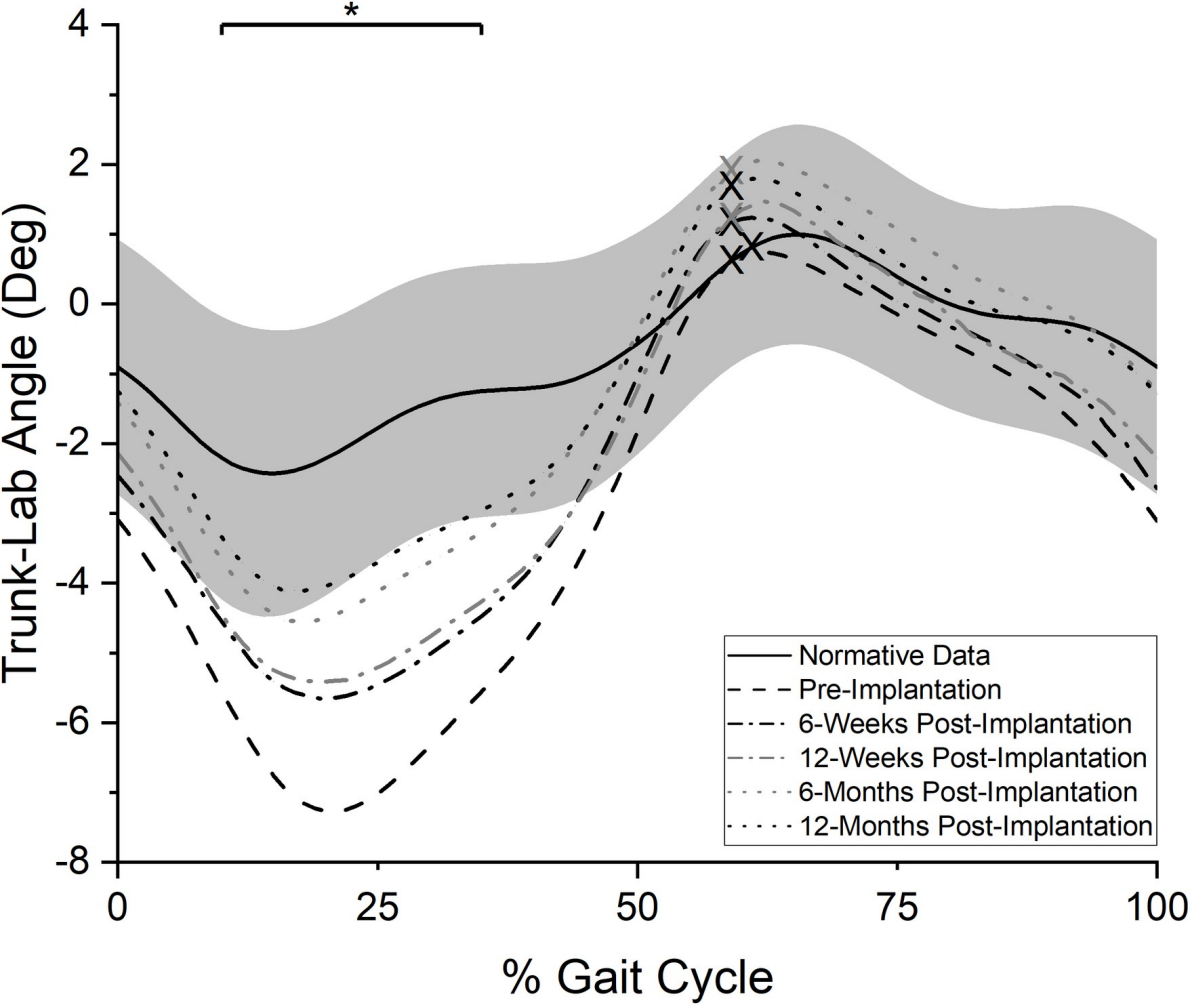
Frontal plane trunk angle. Averaged, normalized frontal plane trunk angle values over the prosthetic side gait cycle (0% = POP side heel strike, 100% = subsequent POP side heel strike). “X” marks represent the mean toe-off event for each curve. Positive values indicate ipsilateral shoulder elevation relative to the lab space, while negative values indicate ipsilateral shoulder declination relative to the lab space. Solid black line indicates reference values for individuals without TFA while grey band indicates plus or minus one standard deviation of the reference data. *: period of statistically significant difference in pre-implantation test values from reference data (p = 0.01).

Trunk movement pre-implantation was characterized by a large ipsilateral trunk lean during stance on the prosthetic limb (Fig 4). This pattern was statistically different from reference data during mid to early terminal-stance (10–35% of the gait cycle, p = 0.01). The ipsilateral trunk lean during stance was decreased at 6-weeks post-implantation and the overall pattern of the trunk was not significantly difference from reference values during any point of the gait cycle. The lack of deviations from reference values was maintained at the 12-month post-implantation visit. However, neither the change between the pre- and 6-week post-implantation nor pre- and 12-month post-implantation tests reached statistical significance (both p>0.10).

#### Trunk-pelvis angle

**Fig 5:**
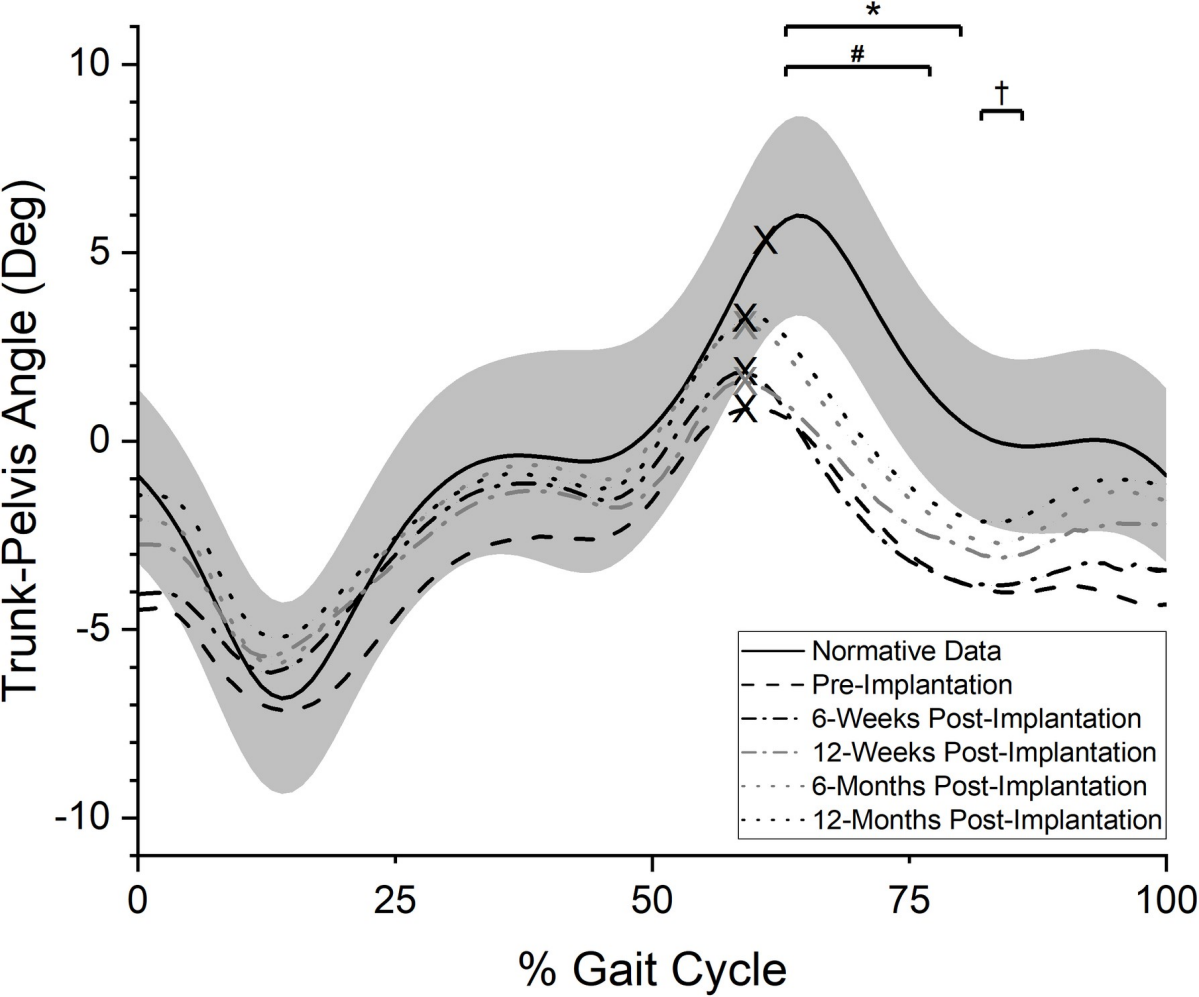
Frontal plane trunk-pelvis angle. Averaged, normalized frontal plane trunk-pelvis angle values over the prosthetic side gait cycle (0% = POP side heel strike, 100% = subsequent POP side heel strike). “X” marks represent the mean toe-off event for each curve. Positive values indicate ipsilateral shoulder elevation relative to pelvis (increased trunk-pelvis angle), while negative values indicate ipsilateral shoulder declination relative to the pelvis (decreased trunk-pelvis angle). Solid black line indicates reference values for individuals without TFA while grey band indicates plus or minus one standard deviation of the reference data. *: period of statistically significant difference in pre-implantation test values from reference data (p = 0.03); #: period of statistically significant difference in 6-week post-implantation test values from reference data (p = 0.04); †: period of statistically significant difference in 12-month post-implantation test values from reference data (p = 0.07).

Pre-implantation, participants demonstrated an ipsilateral lean of the trunk relative to the pelvis during stance on the prosthetic limb, but a more orthogonal position during swing (Fig 5). This trunk-pelvis movement pattern was similar to that of reference values during stance but was statistically different during initial and mid-swing (63–80% of the gait cycle, p = 0.03). A similar pattern to pre-implantation angles during stance was noted at the 6-week post implantation visit with a statistically significant deviation from reference values during initial and mid-swing (63–77% of the gait cycle, p = 0.04). This pattern was not statistically different than the movement pattern observed pre-implantation (p>0.10). Comparison of the 12-month test session data to the reference data revealed a statistically significant difference for a small range of values during swing (82–86% of the gait cycle, p = 0.07). Comparisons between 12-month post-implantation and pre-implantation values for the trunk-pelvis angle did not reach statistical significance (p>0.10).

## Discussion

Prior research suggests sagittal plane movement patterns improved after BAP implantation in those with TFA compared to when the individual used a conventional socket and suspension system [12, 16]. While the current study did not reveal significant differences between frontal plane movement patterns across the three time points analyzed (pre-, 6-weeks post-, and 12-months post-implantation), the results did suggest a reduction in deviations from reference patterns at the 12-month post-implantation visit. Therefore, our results partially support the hypothesis that a BAP facilitates more normalized frontal plane walking patterns.

Before BAP implantation, the individuals in the current study demonstrated differences in frontal plane movement patterns from reference values in line with those reported in prior work, including widened step width, increased hip abduction, and increased ipsilateral trunk lean during single limb support on the prosthetic limb [17, 18]. This compensatory pattern is thought to aid stability over the prosthetic knee and ankle components [28, 29], reduce pressure on the mediolateral aspect of the residual limb within the socket and improve comfort [3, 30], stabilize the residual limb against the lateral wall of the socket, and/or decrease muscular work by a weak or mechanically disadvantaged hip abductor [31, 32]. Further, an ipsilateral pelvic hike and compensatory decrease in trunk-pelvis angle during prosthetic limb swing was also observed among the current participants pre-implantation. This pattern may be an effort to compensate for hip abductor weakness or to compensate for the soft tissue movement between the bone and the socket. It may also help counteract the nearly 2 cm of limb-socket axial displacement that occurs with conventional sockets to increase foot clearance during limb advancement and decrease the risk for a stumble or fall [33, 34].

Changes at the 6-week exam were of interest as eliminated or significantly diminished frontal plane deviations by the first post-implantation test could have suggested the improvements were more likely related to the implant than other physical rehabilitation related factors. However, the 6-week post-implantation data showed frontal plane movements patterns did not dramatically improve in such a short-term. Analysis of the frontal plane gait parameters at the first post-implantation visit (Figs 2–5) suggests only modest changes from pre-implantation, with a reduction in lateral trunk lean during stance being the largest change immediately after the POP implantation. This lack of change may be related to the time needed for bone and soft tissue healing, muscle strengthening due to post-amputation atrophy, and the potential need to “unlearn” habitual pre-implantation gait deviations. Similar to the phenomenon demonstrated in those after total knee replacement [35], a habitual gait deviation may persist even after the implant provided a reduction in pain or increase in subjective function. An implication of the current results is that it may be unrealistic to expect a rapid normalization of gait patterns immediately post-implantation. Instead, the gradual reduction in frontal plane deviations relative to reference values by the 12-month post-implantation test suggests more normative gait patterns take longer to achieve. The individual participant data (S1 File) may also support this interpretation as a majority appeared to respond in a manner consistent with the group level progressive reductions in frontal plane gait deviations. However, further research is necessary to test this hypothesis.

An increase in functional strength of the prosthesis side hip muscles may be one potential reason for finding the BAP device promoted normalization of frontal plane movement patterns. Those with TFA commonly present with weakness and atrophy in the hip abductor muscles [31, 32], and hip abductor weakness is associated with the same stance-phase trunk and hip frontal plane deviations demonstrated in the current study [31]. The direct connection between the residual limb and the rest of the prosthetic limb via the BAP implant may have increased use of the muscle group during activity and ultimately stimulated muscle hypertrophy. Prior research corroborates this hypothesis as results showed that an individual with an OI implant developed increased hip abductor volume at both 6-months and 12-months post-implantation [36]. It should be noted that rehabilitation after amputation often includes exercises that target hip muscles. Those exercises could also contribute to increases in hip abductor strength and subsequent reductions in gait kinematic deviations. While all study participants received a minimum of 2-weeks of outpatient physical therapy after the BAP surgeries and prosthetic fitting, the exact number and duration of therapy sessions after the implantation procedures were not collected, and no individual in the study received regular prescribed physical therapy or gait training past their 6-month visit. Furthermore, though the data were collected prospectively, the results were not shared with clinicians treating the participants. Any therapeutic exercises or gait training was provided without specific knowledge of pre-existing gait deviations identified from the data collected. Taking together, the reduction in gait deviations from reference values by the 12-month post-implantation tests were seemingly related to a cumulative effect of continued use of the implanted device rather than the limited rehabilitation training the individuals received. Ultimately, the BAP may not immediately provoke changes in the individual’s movement patterns, but instead provide a condition in which the normalization of frontal plane gait movements can occur. Prior work examining other BAP implant systems suggests activity levels increase after transition to a BAP [37], supporting the notion that increased activity overall likely contributed to increased muscle strength and an improved gait. While activity levels were not analyzed as part of this kinematics study, improvements for the group in mobility-related outcomes [19] suggest the activity levels may have increased.

Despite the reductions in frontal plane deviations from reference values, not all were eliminated at the 12-month post-implantation visit. The remaining differences may have persisted because the individual’s prosthetic limb did not fully compensate for the functional loss of their biological knee, foot and ankle, regardless of a direct connection provided by the BAP. For example, our results showed step width narrowed slightly over time but remained well above norms [38]. Prosthetic knees and ankles do not provide the same frontal plane control as biological structures, and as such the widened base of support may have served to maintain stability. Furthermore, the remaining pelvis and trunk-pelvis deviations during prosthetic limb swing may have persisted because the prosthetic ankle did not provide active dorsiflexion and may have hindered foot clearance even without limb-socket distraction.

Though a more normalized frontal plane movement pattern was demonstrated at the 12-month post-implantation test relative to the reference data, there was no statistically significant change in the frontal plane measures between the pre- and 12-month post-implantation tests. An explanation for this finding may be the functional level of the participants. Though these individuals elected to transition away from a conventional suspension system due to socket-related issues, they were relatively high-functioning prior to the BAP implantation as evidenced by both their high K-level and SSWS (Table 1). As a group, the SSWS of the participants was higher than values reported for others with TFA [39], was fast enough to approach normative values for those without amputation [40], and did not display a significant difference between from pre-implantation and 12-months post-implantation values. The kinematic differences between these individuals before BAP implantation compared to reference values may be minimal compared to other, less functional individuals. This may have created a floor effect for their potential normalization after device implantation. Regardless, small changes in frontal plane kinematics may still be meaningful. For example, a 3–4 degree difference in lateral trunk lean coincided with significantly increased trunk compression forces in individuals with limb amputation compared to individuals without limb amputation [41, 42]. The compressive forces are theorized to lead to back pain and dysfunction, and thus a small reduction in frontal plane trunk kinematics magnitude could conceivably change spinal joint loading and alter clinical outcomes. In this context, the current results suggest transitioning to a BAP could have other benefits (i.e., prevention of back pain) that have not yet been explored.

There are several other points to consider when interpreting and generalizing the study’s results. First, the sample size within this preliminary study was limited by constrained design. This FDA EFS only allowed for 10 participants of which 8 individuals had suitable data for analysis, including one individual whose 6-month post-implantation data was utilized in place of 12-month post-implantation data. The small sample size limits the ability to examine differences between other time points within this group. The participants were also prescribed different prosthetic knee or ankle componentry, and provided alignment changes based on their individual clinical needs. Moreover, the prosthetic device changed for some individuals during the 12-months (reasons for changes included replacement of components needing service, and requests for a waterproof knee or more flexible feet). It is unknown what affect these changes may have had on an individual’s gait patterns. Additionally, all participants in this study underwent amputation due to trauma, and as such, may not represent the response of those who may have lost limbs due to vascular dysfunction or diabetes. However, as implantation with a BAP is not widely performed yet in those with limb loss due to vascular conditions, the individuals in this study are more representative of those who actually undergo BAP implantation. Moreover, other factors that could have affected gait, such as pain levels, were not assessed in this study and the potential effects of these factors on the current results is unknown. This data also only encompasses the first year after BAP implantation. While we hypothesize that further normalization in gait patterns may occur later than one-year post-implantation, the data to assess this hypothesis was not collected as part of the study. Continued follow-up is planned to assess the long-term response of the participants to the BAP implant. Finally, for comparisons to normative values, we used a reference group of young, healthy individuals without limb amputation walking on a treadmill. While some research has previously found kinematic differences between treadmill and overground walking, the differences in frontal plane motion of the hip, trunk, and pelvis previously found are much smaller than the deviations described here [43–45]. Therefore, the differences found in the current study are more likely to be a due to true difference between groups rather than from the walking modality.

## Conclusion

This study is the first to describe frontal plane gait kinematics after a BAP implant in those with TFA. While comparisons between frontal plane movement patterns pre-implantation and 12-month post-implantation were not statistically different, deviations from reference values that occurred during both prosthetic side stance and swing were gradually reduced or eliminated over the first year after implantation with the POP implant system. These results add to the literature suggesting that transition to a BAP may aid in normalization of gait parameters in those with TFA that fail to tolerate traditional socket and suspension systems.

## Supporting information

S1 File Individual participant results. (DOCX)

S2 File (DOCX)

S3 File (DOC)

S1 Dataset (XLSX)
