# Supplementary material for: Changes in frontal plane kinematics over 12-months in individuals with the Percutaneous Osseointegrated Prosthesis (POP)
Source: PLoS One. 2023 Feb 22;18(2):e0281339. doi: 10.1371/journal.pone.0281339 (PMC9946262; doi:10.1371/journal.pone.0281339)
Supplement: S1 File — (DOCX) [file pone.0281339.s001.docx]

**S1 File. Graphs of individual Participants’ frontal plane kinematic patterns at the pre-implantation, 6-weeks post-implantation, and 12-months post-POP implantation sessions.** Dashed lines indicate pre-implantation, dot-dash lines indicate 6-weeks post-implantation, and dotted lines indicate 12-months post-implantation visit values. “X” marks denote average toe-off event. For Participant 8, the 6-month post-implantation values are used in place of the 12-month values.

| **Hip Adduction Angle** | | | |
| --- | --- | --- | --- |
| **Participant 1** | **Participant 2** | **Participant 3** | **Participant 4** |
| 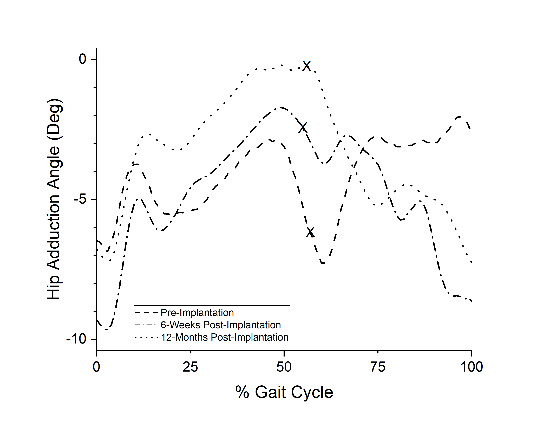 | 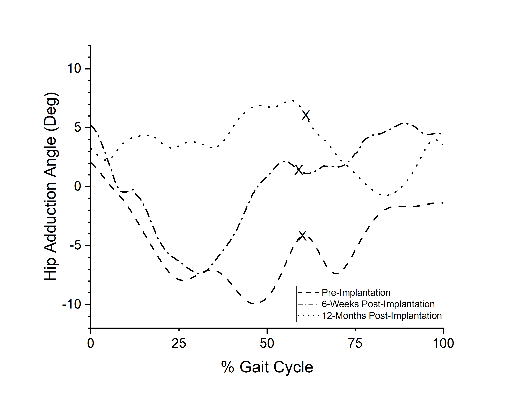 | 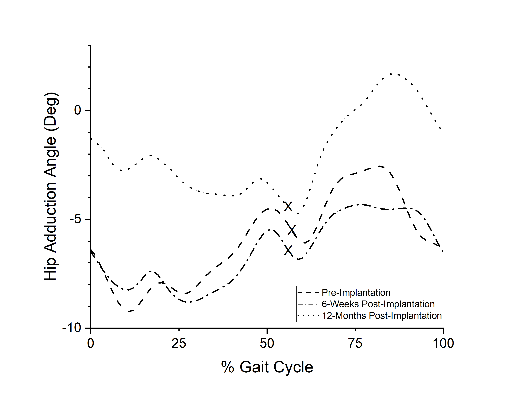 | 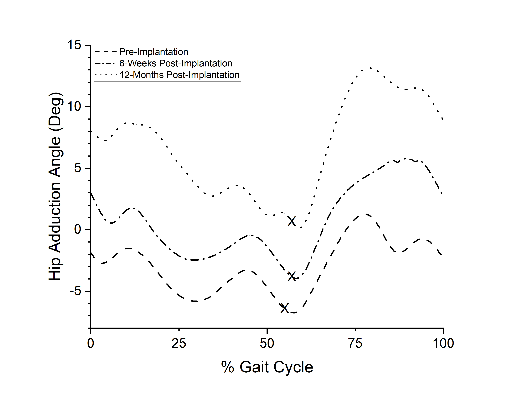 |
| **Participant 5** | **Participant 6** | **Participant 7** | **Participant 8** |
| 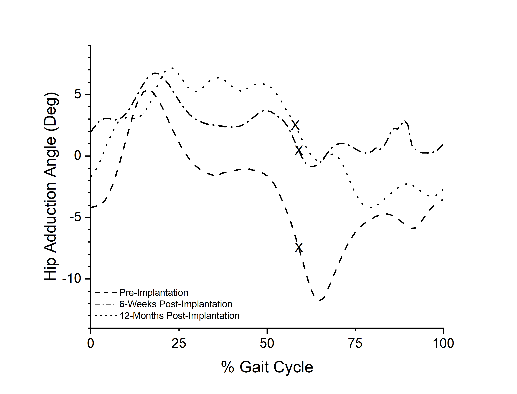 | **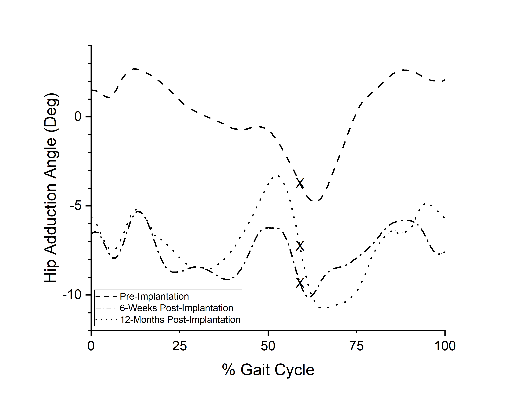** | 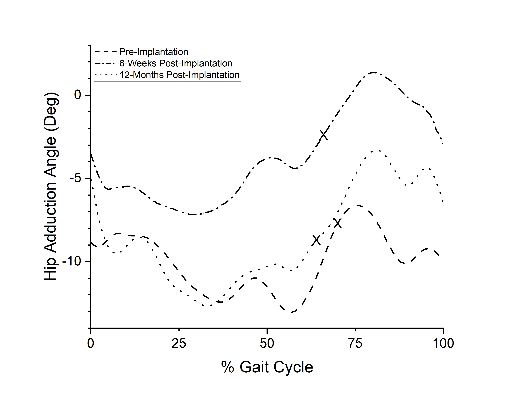 | 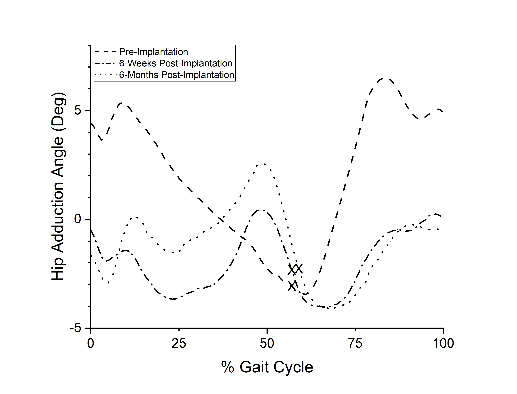 |

| **Pelvis-Lab Angle** | | | |
| --- | --- | --- | --- |
| **Participant 1** | **Participant 2** | **Participant 3** | **Participant 4** |
| 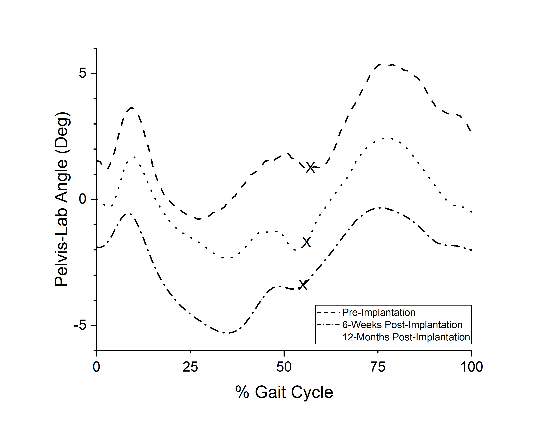 | 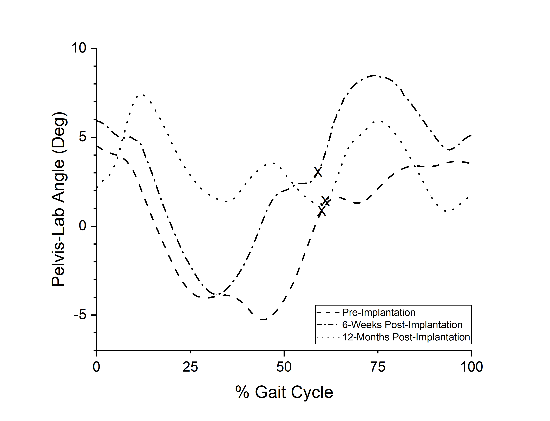 | 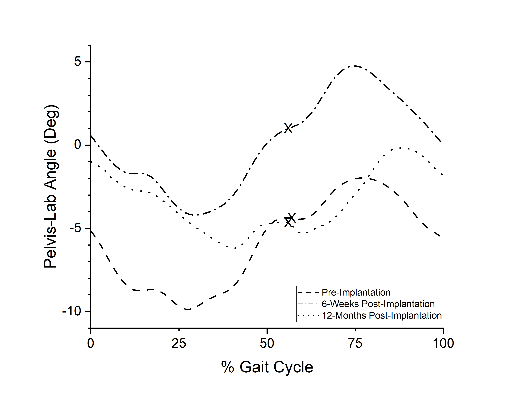 | 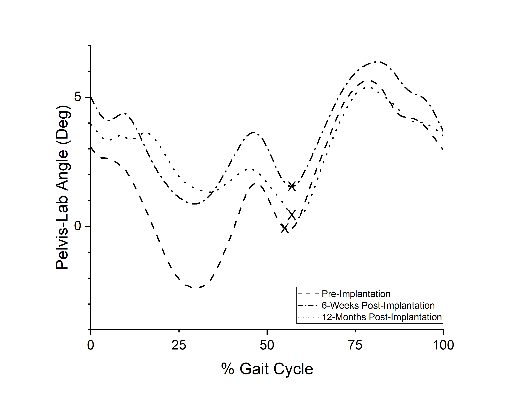 |
| **Participant 5** | **Participant 6** | **Participant 7** | **Participant 8** |
| 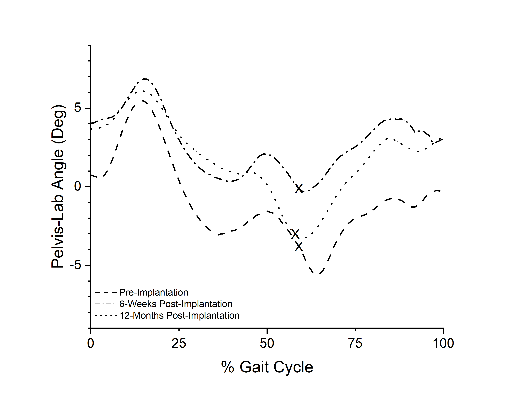 | **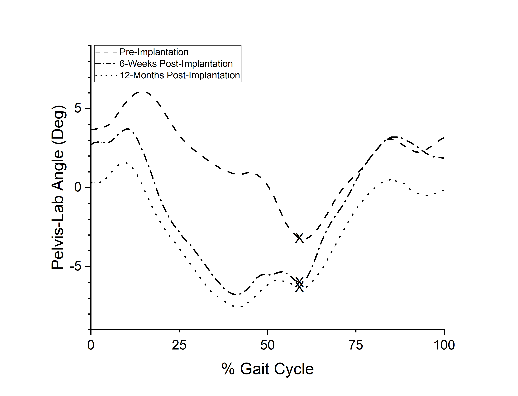** | 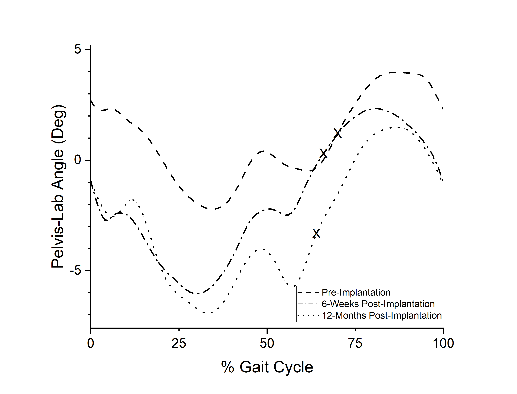 | 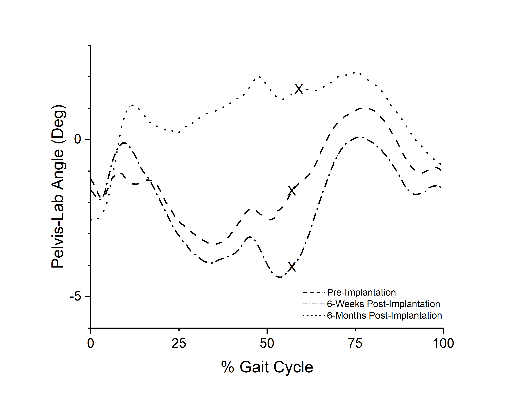 |

| **Trunk-Lab Angle** | | | |
| --- | --- | --- | --- |
| **Participant 1** | **Participant 2** | **Participant 3** | **Participant 4** |
| 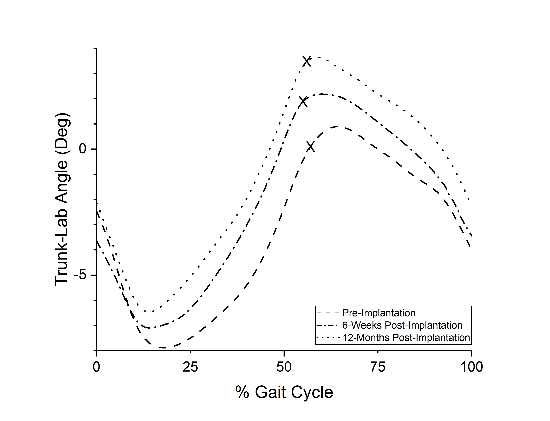 | 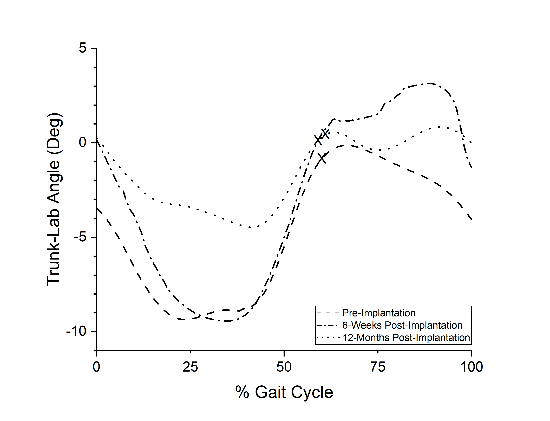 | 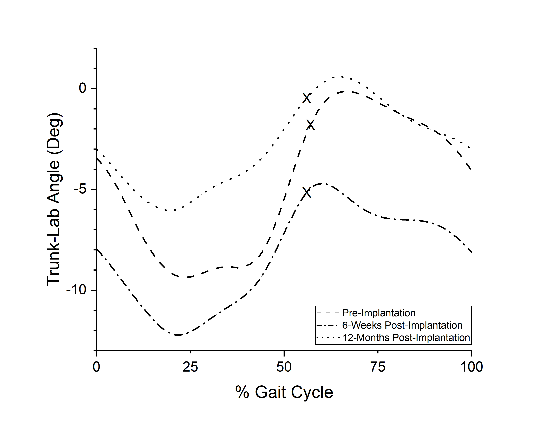 | 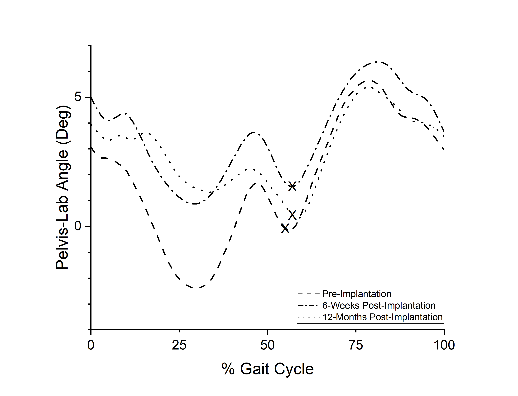 |
| **Participant 5** | **Participant 6** | **Participant 7** | **Participant 8** |
| 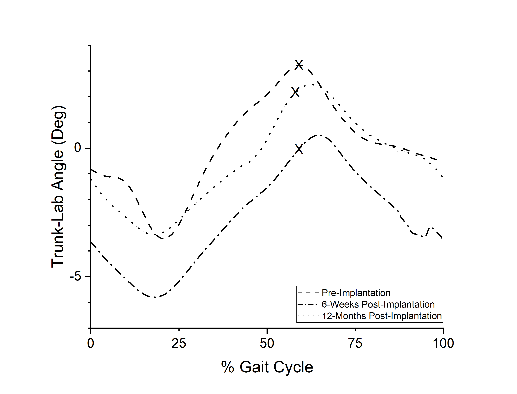 | 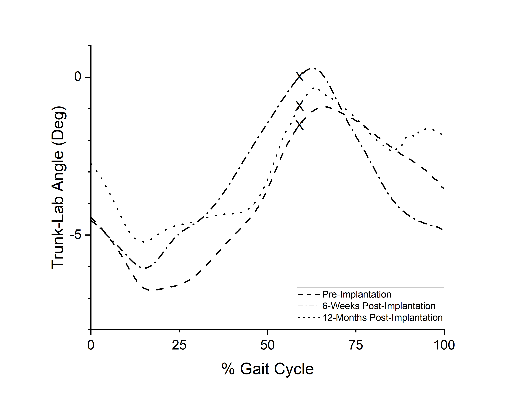 | 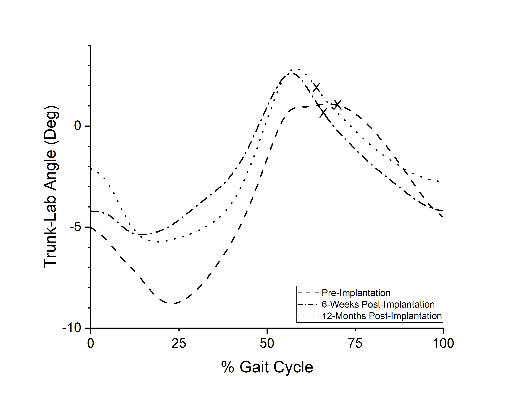 | 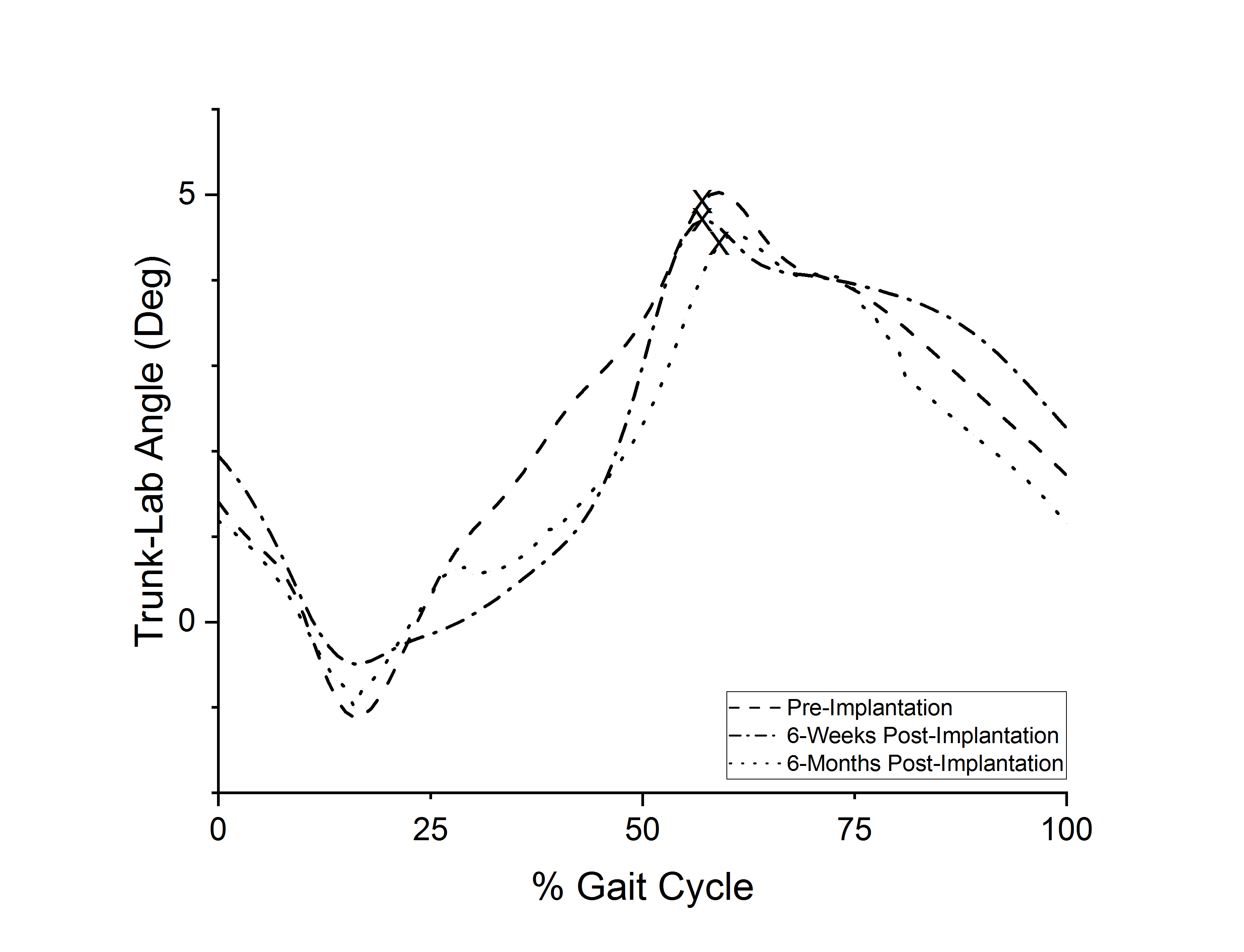 |

| **Trunk-Pelvis Angle** | | | |
| --- | --- | --- | --- |
| **Participant 1** | **Participant 2** | **Participant 3** | **Participant 4** |
| 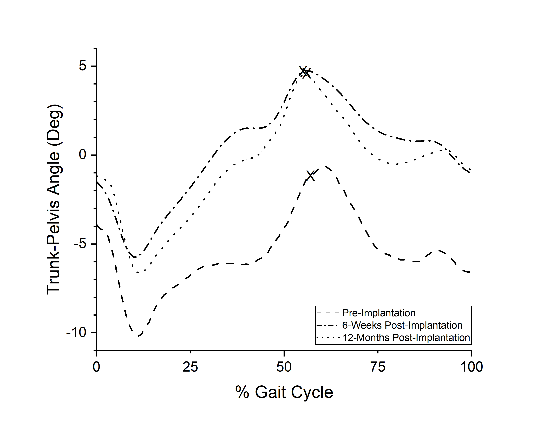 | 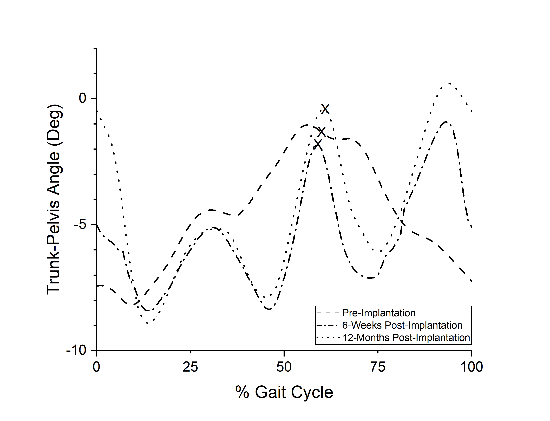 | 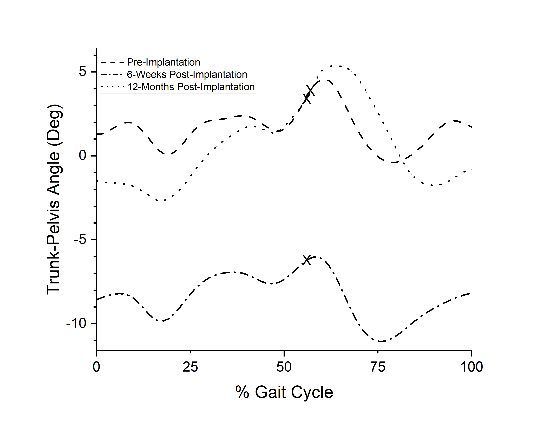 | 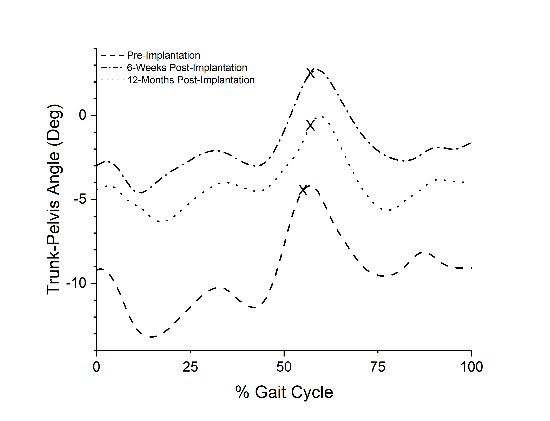 |
| **Participant 5** | **Participant 6** | **Participant 7** | **Participant 8** |
| 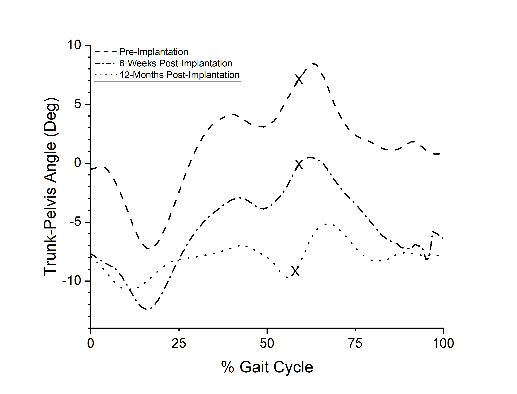 | 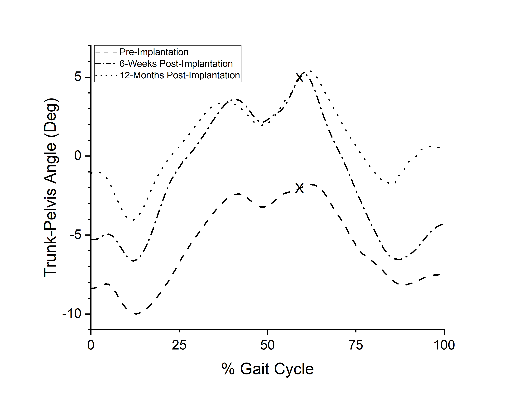 | 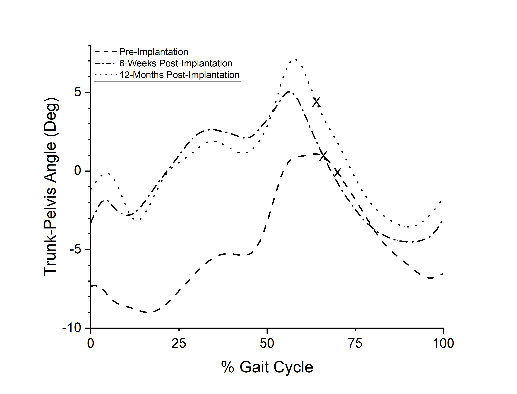 | 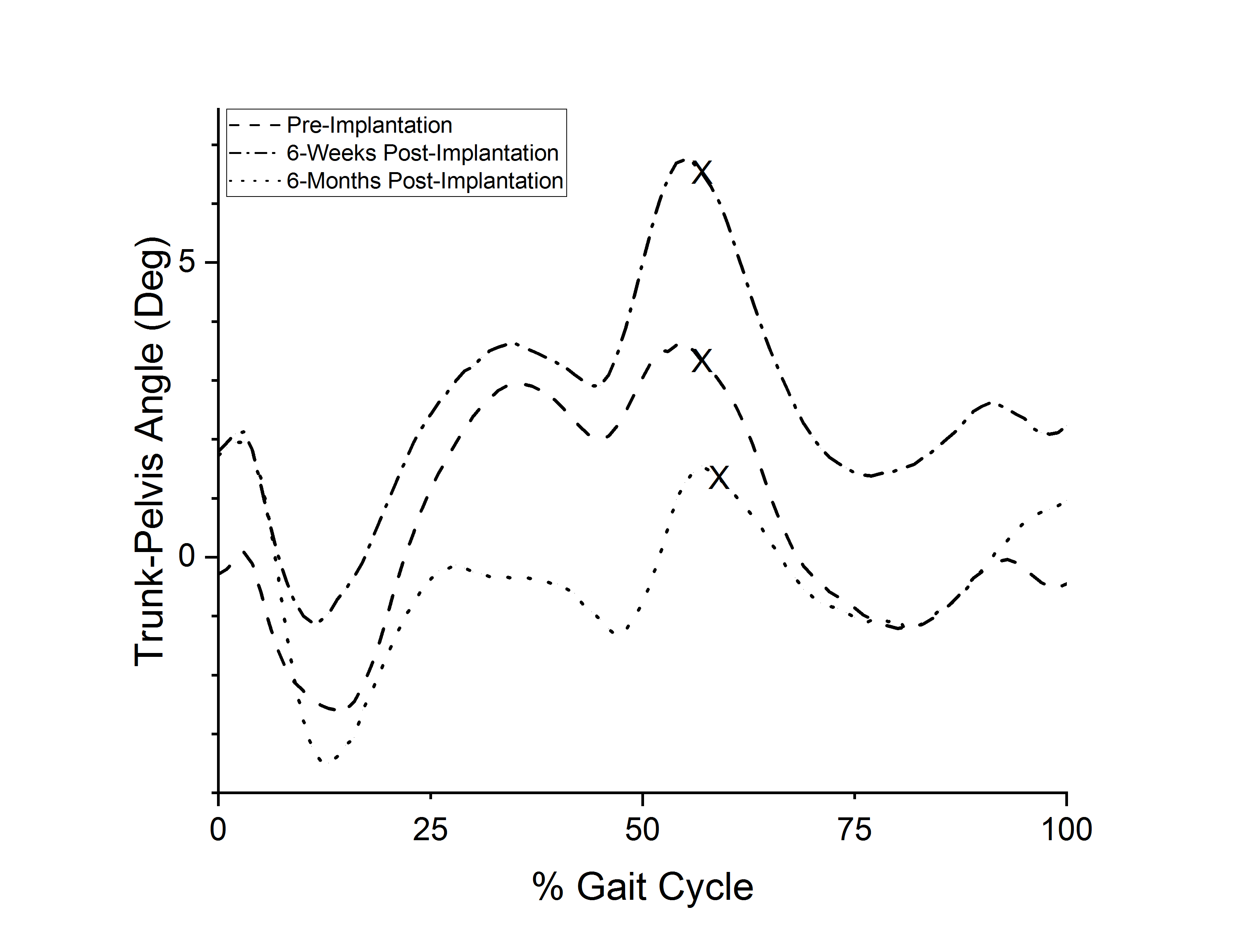 |
