# Supplementary material for: Changes in frontal plane kinematics over 12-months in individuals with the Percutaneous Osseointegrated Prosthesis (POP)
Source: PLoS One. 2023 Feb 22;18(2):e0281339. doi: 10.1371/journal.pone.0281339 (PMC9946262; doi:10.1371/journal.pone.0281339)
Supplement: S2 File — (DOCX) [file pone.0281339.s002.docx]

| **Safety Study of Percutaneous Osseointegrated Implants for Prosthetic Attachment** |
| --- |

**Protocol Summary**

|  |
| --- |

| **IRB Approval Date of Current Version:** | 7/26/2020 |
| --- | --- |
| **University of Utah IRB #:** | IRB_00082763 |
| **Sponsor:** | VA REHABILITATION RESEARCH AND DVLPMNT |
|  |  |
| **Principal Investigator:** | Sarina Sinclair |
| **Internal Staff and Sub-Investigators:** | \| \| **Site Name** \| **Staff Names** \| \| --- \| --- \| \| Veterans Affairs SLC Health Care System (VAMC) \| Sarina Sinclair \| \|  \| Jeremy Gililland \| \|  \| Jayant Agarwal \| \|  \| Bart Gillespie \| \|  \| K. Bo Foreman \| \|  \| Jacob Smith \| \|  \| James Beck \| \| \| --- \| --- \| --- \| --- \| --- \| --- \| --- \| --- \| --- \| --- \| --- \| --- \| --- \| --- \| --- \| --- \| --- \| |

*This document was created using the ERICA Online System at the University of Utah. The document is created from study information approved by the IRB on the date listed above. Any alteration to the original content of this document may not be considered to represent the study as approved by the IRB.*

**Background and Introduction**

|  |
| --- |
| Over the past eight years, researchers at the University of Utah have worked to develop a Percutaneous Osseointegrated Prosthetic (POP) device based on the total hip paradigm in terms of how the endosteal bone of the femur becomes attached to an osseointegrated femoral stem. The Utah device also incorporates some of the characteristics of the skin interface of the deer antler, wherein skin and underlying soft tissue is sealed to the porous structure of the bone around the base of the antler .[^23^](#_ENREF_6) By using what is known about these observed biological events the Utah team developed a device that, in the ovine model, achieved rapid bone in-growth and strength of attachment while providing a “soft tissue seal” around the porous structure of the device.[^24^](#_ENREF_7) This implant system avoided deep and superficial infection by preventing bacteria from entering the intramedullary canal of the implanted bone through the skin stoma over a 1-year period.[^24^](#_ENREF_7) After securing over $5M in Veterans Administration (VA) and Department of Defense grants designated for the advancement of amputee care for wounded warriors and veterans, the Utah team set out to study various means to develop a biobarrier at the interface between the soft tissue and the percutaneous post of the OI endoprothesis. DJO Surgical’s new proprietary titanium P^2^ porous coating (510[k] number K081678) was used to achieve both the bone in-growth and a soft tissue biobarrier material on the implant. The Utah team was able to support the hypothesis that implant osseointegration and a skin barrier to prevent infection could be established in a sheep amputation model. In this large animal translational study, involving over 86 sheep implanted with a POP device, no infections were found up to 1-year after surgery in the P^2^ experimental group compared with a 25% infection rate in the smooth surface control group without the P^2^ biobarrier.^9^ In a follow-up study two-year study animals,which had primary skin-attachment to the P^2^ surface, also maintained an infection-free interface for two years (n = 5 out of 6 animals) in spite of continued epithelial downgrowth. Mechanical strength of the implants was also demonstrated as there was no implant breakage, implant loosening or P^2^ coating failure in either the one- or two-year sheep studies.^10,11^  For the past three years DJO Surgical has worked with the Utah research team, engineers and surgeons to design a new human POP device based on this preclinical work with the goals to limit or eliminate infection and to provide a mechanically stable implant. This new human device design is based on extensive radiographic and CT studies of human cadaveric femurs dimensions to determine the proper POP implant sizing to best accommodate the biomechanical length of the residual femur. Biomechanical studies were conducted in cadaveric femurs at various biomechanical lengths to develop instrumentation, optimize implant sizing and to determine the optimal length of porous coating to be used for initial interlock with the endosteal bone surface.  In spite of these encouraging large animal translational studies and close attention to the results of implantation of such devices in the European amputee populations, we feel there are some aspects of soft tissue management, device design and surgical procedural techniques in humans that require additional refinement.  The surgical management of the new skin seal is in need of further investigation, in particular to determine whether dermal or subdermal tissue attachment to the implant yields the best results.  Dermal ingrowth has been observed in the sheep study, while subdermal attachment may allow the skin to more naturally heal around the post in humans, this cannot be clarified in the ovine model.^9^ Sheep skin may not be an ideal model for human implant-skin integration/wound healing due to the differences in hair follicle density and that wool has a waxy coating, lanolin, that repels water and may protect the skin by limiting bacterial growth around the implant. Also, growth factors expressed during wool follicle development and the different stages of seasonal wool growth differ from what occurs in human hair growth. Skin vascularity, the epidermis and the subdermal tissues also differ markedly between the two species. Importantly, the metacarpal region of the sheep limb used in the preclinical studies has no muscle and relatively little subcutaneous soft tissue exposed to the POP device. The presence of muscle and adipose tissue in the human residual limb is substantially different from the situation in the sheep model and may intensify the biocompatibility response, which cannot be studied with any available animal models. Recent design changes to the exiting post in the German system, and changes in surgical technique and soft tissue preparation for Stage Two implantation, have substantially reduced the incidence of infection in German patients but the precise reason for this reduction is still speculative.[^25^](#_ENREF_8) Although, the European stem designs have failed to address the problem of stress shielding of bone.[^26^](#_ENREF_9) This results in bone resorption and compromises the longevity of the implant and may cause excessive bone removal if revision is required. The Branemark OPRA designs require up to 18-months of partial weight-bearing to become safely osseointegrated due to the mechanical instability of the threaded design, delaying full loadbearing ambulation.  Based upon our knowledge of the European design limitations and our translational animal research we have developed a device design that allows rapid and strong osseointegration, almost immediate weight-bearing, endloading and distal osseointegration to avoid proximal stress shielding to allow easier and safer revision with limited bone loss, and the ability to limit and avoid infection.  Because appropriate nonclinical tests are unavailable, a limited human clinical study of this device design is necessary. Additional nonclinical testing is unlikely to provide the insights necessary to further the development of a safe and efficacious device.  Traditionally, wound microbiotas have been characterized by culture based assays. Cultures, however, account for only 3% of the bacterial strains actually present. Bacterial cultures, because they are designed to select for specific pathogens, are unable to grow and recognize most of the other resident skin microbiota. These non-molecular techniques do not allow a full understanding of the complexity of stomal colonization in both the diseased and healthy states. Wound healing involves a complex process with many overlapping stages: coagulation, inflammation, cell proliferation and migration, and finally tissue remodeling with wound closure within 3-14 days. Understanding the relationship between the microbiome and modulating the cycle of inflammation produced by microbial colonization and/or infection may help improve treatment strategies generally, unrelated to the current device. Comparing the profiles of local and systemic inflammatory biomarkers may provide valuable information regarding the state of health of the skin. Because different cytokines can have overlapping biological functions, as well as regulate production of other cytokines, the ability to simultaneously analyze a large set of cytokines within a localized environment (such as a site of inflammation) can be more valuable than detecting the levels of individual cytokines (particularly if only small volume test samples are obtained). Collecting multiple samples over time will also allow for longitudinal studies of cytokine profiles within a single patient.  These additional samples provide the opportunity to collect valuable information on multiple microbiomes involved in the wound healing response rather than specific cultures for individual microbiota It is also hoped that this broad spectrum analysis will provide more detailed information both over time and within subject on the wound healing treatment strategy for the full Investigational Device Exemption Study proceeding this Early Feasibility Study. |

**Purpose and Objectives**

|  |
| --- |
| The purpose of the clinical study is to characterize the efficacy and safety of a Percutaneous Osseointegrated Prosthesis (POP) device that can provide a satisfactory platform for direct skeletal attachment of prosthetic limbs.  The primary objective for this study is   - To determine the efficacy and safety of this novel device as a docking system for prosthetic limbs following transfemoral limb amputation.   Secondary objectives include:   - To evaluate the surgical staging and techniques used in the procedure relative to dermal and subdermal soft tissue attachment. - To assess the device design relative to potential modification that may offer an improved long-term safety and efficacy profile i.e. stress shielding in host bone. - To evaluate the mechanical durability of the implant construct through rates of implant fracture, component loosening and porous coating failure. - To identify and evaluate any device-related adverse events such as aseptic mechanical loosening of the bone implant interface. - To define the rehabilitation process and assess recipient acceptance and functional use of the device - To determine if there is a correlation with the microbiota pattern. - To evaluate the local and systemic inflammatory biomarkers over time. |

**Study Population**

|  |
| --- |
| **Age of Participants:** 18+  **Sample Size:**   \| At Utah: \| 10 \| \| --- \| --- \| \| All Centers: \| 10 \|   **Inclusion Criteria:**   \| \| A potential subject will be included in the study if he or she meets all of the following inclusion criteria:   \| - - Is a US military veteran with transfemoral amputation that is not a result of dysvascular disease.   - Amputation occurred at least 6 months prior to consent   - Is at least 18 years of age or older.   - Has previously used or is currently using a “socket suspension technology” prosthesis.   - Has, in the consensus of the investigator, surgical team and clinical psychologist, no physical limitations, addictive diseases, or underlying medical conditions including tobacco use (continued testing for tobacco use will be performed at screening) that may prevent the subject from being an appropriate study candidate.   - Is willing, able, and committed to participation in baseline and follow-up evaluations for the entire duration of the study.   - Can provide IRB approved written informed consent to participate.   - The use of non-propulsive, passive microprocessor-regulated devices (C-leg, Rheo, Plie, Elan or simular)   - Agrees not to participate in high levels of physical activity while participating in the study. \| \| --- \| \| \| --- \| --- \| \| \| --- \| --- \| --- \|   **Exclusion Criteria:**   \| \| A potential subject will be excluded from study participation if he/she meets any of the following criteria:   \| - - Is currently on active military duty   - Has experienced systemic bacterial infection or localized infection at the residual limb site within the previous 6 months   - Has had more than 1 limb amputated   - Has a body mass index (BMI) ≥ 30   - Has insulin dependent diabetes mellitus (IDDM) or has adult onset DM with a glycated hemoglobin (HbA_1c)_ > 53 mmol/mol (7.0%) at screening   - Has clinically diagnosed vascular compromise proximal to the surgical site   - Is pregnant at the time of surgery or plans to become pregnant within the first year of follow-up   - Has evidence of recent tobacco use (urine cotinine test > 300 ng/mL [1703 nmol/L]) and is not committed to a smoking-cessation program   - Has renal insufficiency (defined as serum creatinine of ≥ 1.8 mg/dL) or is currently receiving renal dialysis   - Has muscular, neurologic, or vascular deficiencies that may compromise the bone or soft tissue healing of affected extremity   - Has anemia characterized by a hemoglobin of ≤ 11 g/dL   - Is currently on oral anticoagulation (excluding low-dose aspirin for cardiac prophylaxis)   - Has history of immunosuppressive therapy   - Has a history of residual limb skin grafting in the in the surgical field area affecting soft tissue closure that may compromise early soft tissue healing or long term stoma durability   - Has active heterotopic ossification as observed on radiography and as defined by elevated alkaline phosphatase or a hot bone scan   - Has a history of known sensitivities to any materials used in the prosthesis   - Has a severe comorbidity or poor general physical/mental health that, in the consensus of the investigator, surgical team and clinical psychologist, will not allow the subject to be a good study candidate (i.e., other disease processes, mental capacity, substance abuse, shortened life expectancy, vulnerable subject population, history of keloid formation, history of osteomyelitis, etc.).   - Is currently involved in another clinical study in which participation may conflict or interfere with the treatment, follow-up, or results of this clinical study to screening   - Has a history of known sensitivities to any materials used in the prosthesis   - The use of active, propulsive microprocessor knees (Ossur Power Knee, BiOM, or similar) \| \| --- \| \| \| --- \| --- \| \| \| --- \| --- \| --- \| |

**Design**

|  |
| --- |
| \| Prospective Biomedical Intervention or Experiment \| \| --- \|  \| FDA approved Early Feasibility Study:  This will be a first-in-man, nonrandomized, prospective early feasibility study (EFS) using a novel prosthetic device system for direct skeletal prosthetic limb attachment in transferal amputees.  The FDA has granted Dr. Erik Kubiak permission to investigate this device in 10 people as part of an EFS. The information from this study might help us change the design of the implant.  According to the FDA: "Early feasibility studies allow for early clinical evaluation of devices to provide proof of principle and initial clinical safety data. These studies may be appropriate early in device development when clinical experience is necessary because nonclinical testing methods are not available or adequate to provide the information needed to advance the developmental process. As with all clinical studies, initiation of an early feasibility study must be justified by an appropriate benefit-risk analysis and adequate human subject protection measures."(1) \| \| --- \| |

**Study Procedures**

|  |
| --- |
| **Recruitment/Participant Identification Process:**   \| -Any military or VA health care system provider will be used to identify potential participants at both VA and military hospitals throughout the United States  (surgeons, rehabilitation, physical therapy, prosthetics, etc.)  -Informational materials (see documents) will be sent to the following groups: Wounded Warrior Project, the Federal Amputation Interest Group (FAIG) list serv, VA health care system prosthetists, VA health care system physical therapists  -Using the VA Computerized Patient Record System**(**CPRS) a search will be conducted for unilateral transfemoral amputee patients.  -All potential participant charts will be reviewed by Drs. Sarina Sinclair, Ami Stuart and Bart Gillespie using the VA Computerized Patient Record System **(**CPRS).  -Following screening protocol approved under **IRB_00063220,** two investigators will separately review the charts for unilateral transfemoral amputees and only investigators at the Salt Lake City VA will complete inclusion/exclusion case report form (CRF) for the recommended patients using CPRS. Identifiers of patients who meet study inclusion/exclusion criteria will be recorded. Clinical investigators will be provided the CRFs for patients who meet inclusion/exclusion criteria and are potential study participants.  -Following screening protocol approved under **IRB_00063220,**patients will be identified that are eligible for this Early Feasibility Study. When an eligible patient is identified, they will be sent a Early Feasibility Study recruitment letter (see documents) that outlines the study purposes, procedures, risks, and benefits.  -If there is no response to the letter, a follow-up phone call will be made at two and four weeks after the letter mailing date. No further attempts will be made to contact the patient after four weeks. \| \| --- \|   **Informed Consent:**   \| **Description of location(s) where consent will be obtained:** \| \| --- \| \| Consent will be obtained via mail or at the Salt Lake City VA \|  \| **Description of the consent process(es), including the timing of consent:** \| \| --- \| \| Drs. Kubiak, Beck, and Agarwal will recuse themselves from participating in the informed consent process from all potential research participants for this study. Informed consent for all potential research participants will be obtained by a member of the research team who does not have any conflicts of interest related to this research or by an appropriate third party approved by the Individual Conflict of Interest Committee. Using the list of contact information for patients who meet study inclusion/exclusion criteria an investigator will contact the patient via telephone to discuss the study purposes, procedures, risks, and benefits. The patient will be informed of how s/he was chosen to be contacted and the investigator will explain the procedures to follow including travel to the site. S/he will answer any questions the patient has before asking the patient if s/he would be willing to read and sign a consent form to participate in the study. If the patient agrees, a consent form will be mailed with a self-addressed, pre-stamped envelope that clearly explains the screening procedures and travel arrangements. The patient will be given contact information for an investigator that can answer any questions s/he has prior to signing the document. The patient will be asked to return the consent form in person or in the envelope provided and to call the investigator when s/he mails the envelope. The patient will be given three weeks to return the consent form before s/he is contacted via telephone to inquire whether the form was received and/or returned. \|  \| **Requested Waivers/Alterations of Consent:** \| \| \| --- \| --- \| \| Waiver of Informed Consent \| Record review for recruitment \|   **Procedures:**   \| Using the list of contact information for patients who meet study inclusion/exclusion criteria (IRB 63220) and respond to the recruitment letter, an investigator will contact the patient via telephone to discuss the study purposes, procedures, risks, and benefits. If a message is left on voicemail, no personal health information or study details will be recorded. When contact is made, the patient will be informed of how s/he was chosen to be contacted and given information to contact the investigators if they are interested in participation. The investigator will explain the  procedures to follow including travel to the site. S/he will answer any questions the patient has before asking the patient if s/he would be willing to read and sign a consent form to participate in the Early Feasibility Study. If the patient agrees, a consent form will be mailed with a self-addressed, pre-stamped envelope that clearly explains the Early Feasibility Study procedures and travel arrangements. The patient will be given contact information for an investigator that can answer any questions s/he has prior to signing the document. The patient will be asked to return the consent form in the envelope provided and to call the investigator when s/he mails the envelope.  Upon receipt of the signed consent form, the participant will be contacted by an investigator to arrange travel. Travel, housing and per diem will be paid for by the study.  Those who have been judged to be appropriate for the study, and have provided study consent, will undergo two separately staged surgical procedures for preparation and placement of the POP device. They will then receive routine surgical follow-up care followed by a 12-month period of study-related assessments for device efficacy and safety. Evaluations will include determining user acceptance, subject satisfaction with the device, their ability to perform activities of daily living, quality of life evaluations, improvement in gait and mobility, and improvement in pain status.  This study will consist of the following 6 stages:   1. Pre-Op 2. Stage 1 Surgery 3. Post Stage 1 Rehabilitation (4-6 weeks +/- 14 days) 4. Stage 2 Surgery 5. Post Stage 2 Rehabilitation and follow-up (52 weeks) 6. Long term follow-up arm (until expanded study is approved)   All evaluations to be performed at each study stage are detailed in the tables in Attachment Study Procedures. Subjects will be re-evaluated at Pre-op and if approved by the clinical team, will complete the first stage of a 2 stage surgical process within 4 weeks. In 4 to 6 weeks following the initial procedure, subjects will complete a preoperative visit for the second-stage procedure. That second-stage procedure will establish the timing for subsequent follow-up visits. Subjects will be evaluated at 2, 6, 12, 24, 36 and 52 weeks after the second-stage procedure. Subjects will provide written informed consent prior to Stage 1 surgery. At the end of 52 weeks, subjects will be enrolled in the long-term follow-up arm to cover any visits needed for repair or maintenance of the device until the next phase of the study is approved.  **a. Pre-op:** The Pre-op study session will begin two weeks prior to the participant arriving in Salt Lake City, Utah. At this time the participant will be mailed a stepwatch activity monitor and will be asked to wear the activity monitor during waking hours. Data from the stepwatch will be used as a baseline activity measure. Details regarding the stepwatch activity monitor are uploaded under Other Documents. When the patient arrives in Salt Lake City, Utah, s/he will meet with the surgeons, doctors, rehabilitation specialists, social worker and psychologist to complete all pre-operative procedures to include if needed:   - Anterior/Posterior and Lateral X-rays of residual limb (outside standard clinical care) - Ask detailed questions about your medical history. - Perform a routine physical examination. - Draw blood for a drug screen, clinical lab tests, and (if female) a pregnancy test. - Undergo a skin swab and complete a personal hygiene questionnaire - Collect urine for lab tests, including testing for nicotine use. - Answer questions about your experience with your prosthetic, your mood, and your pain. - Take photographs of the amputated limb before and after surgery. - Have you participate in physical therapy and function testing. You will walk on a treadmill while wearing a mask to measure your breathing. We will record your regular stride while walking for six minutes, while standing on one leg, and while moving as quickly as you can through cones set in various shapes.     **b. Stage 1 Surgery:**  Prior to the Stage 1 procedure, subjects will undergo a physical examination including medical history and clinical lab testing including serum drug screening. They will then undergo the first stage of a two stage surgical process; the stages being spaced approximately four to six weeks apart. Stage 1 surgery will consist of soft tissue revision of the residual limb including myodeses and optimization of the skin/subcutaneous fat envelope. Residual limb revision and bone preparation (broaching) is followed by implantation of the endoprosthetic portion (femoral stem) of the device by the orthopaedic surgeon. The wound is then closed. Any skin samples collected during surgery, that would otherwise be discarded, will be collected and analyzed for pro-inflammatory markers. Postoperatively, subjects will be managed according to the standard of care for above-knee amputations, including anteroposterior (AP) and lateral xrays. On post-op Day 3 +/- 7 days, a skin swab, clinical laboratory testing, and personal hygiene questionnaire will be collected. Subjects will be discharged approximately 4 to 5 days (+/- 7 days) after surgery but will be followed after 4 to 6 weeks (+/- 14 days) for suture removal. Before they are discharged, minimum functional requirements must be met. Additional therapy and/or inpatient rehabilitation will be provided by a study physical therapist as needed to meet the following requirements:   1. Independent bed mobility 2. Independent transfers: bed, chair, wheelchair, toilet, shower, and car 3. Independent ambulation with appropriate assistive device (underarm crutches, forearm crutches, front wheel walker) >200 feet 4. Independent with activities of daily living including toileting and showering while maintaining incision dressing integrity 5. Independent wheelchair propulsion 6. Independent with incision care, dressing changes, and management of residual limb protection device 7. Independent prone positioning   **c. Post Stage 1 Rehabilitation (4-6 weeks)**  Subjects will be instructed not to wear a socket prosthesis or perform residual limb weight-bearing for the first 4 to 6 weeks (+/- 14 days) after discharge. During this period, subjects will be given a physical therapy home conditioning program that will be based on preoperative fitness, function, and goals. Components of this program will include the following:   1. Aerobic conditioning program: upper-body exercise, wheelchair propulsion, body weight circuit, Thera-band circuit 2. Range of motion (ROM) and flexibility program: hip extension for both lower extremities; knee flexion/extension, and ankle flexion/extension for the contralateral limb. 3. Strength program: abdominal/lumbar stability, lower extremity strength, upper extremity strength, body weight resistance, Thera-band resistance, weight resistance   Any ROM, strengthening, or activity restrictions will be clarified and reviewed to assure compliance in the home environment.  **d. Stage 2 Surgery**  During the preoperative phase of Stage 2, at 4-6 weeks (+/- 14 days) following the Stage 1 procedure, the subject will again be evaluated for study eligibility relative to inclusion and exclusion criteria. Subjects will undergo a physical examination including imaging, querying for adverse events, skin swab and personal hygiene questionnaire and clinical lab testing including serum drug screening, and a physical therapy evaluation. Skin imaging and grading will be performed. The percutaneous post will be implanted during Stage 2 surgery. Prior to implantation of the percutaneous post, the residual limb and incision will be checked for any signs of infection and evidence of improper wound healing including swelling, pain, incision separation, ecchymosis, scaling, serous drainage, or delayed capillary refill time (greater than 2 second refill time). The surgical procedure, to attach the percutaneous post of the device, will consist of coring a channel (stoma) through the 2 cm of overlying skin and subcutaneous tissue down to the device. The Stage 1 protective cap will be removed, the female Morse taper cleared of tissue fluids and the connecting percutaneous post attached. Any skin samples collected during surgery, that would otherwise be discarded will be collected and analyzed for pro-inflammatory markers. Postoperatively, subjects will again be managed according to routine postoperative standard of care for amputees. Subjects will be transferred to the PACU where AP and lateral x-rays will be obtained. On post-op day 7 (+/- 7 days), skin swap, clinical laboratory testing, and personal hygiene questionnaire will be collected. If local exudates (any drainage of serous fluid from the stoma) are present at any study timepoint after stage 2 surgery, additional swabs may be taken. Special attention will be given to monitoring the skin/implant interface for approximately 2-weeks after surgery before considering discharging the patient. The interface will be checked for any signs of infection and evidence of proper healing which includes degree of: swelling, pain, incision separation, ecchymosis (bruising), scaling, serous drainage, or greater than two second capillary refill time prior to discharge.  Following the stage 2 procedure, the subject’s exoprosthesis components will be fit and aligned to the POP device by the research study prosthetist.  The same prosthetic knee and foot components that the subject used in their pre-operative prosthesis will be used following placement of the POP device.  Once fitting and proper alignment of the external prosthetic components is completed, the subject will begin their Post Stage 2 Rehabilitation physical therapy program for prosthetic training.  For approximately the first 7-10 days following stage 2 surgery, the subject will be restricted to performing only static weight-bearing activities in standing with the exoprosthesis attached to the POP device.  The subject will be restricted to partial weight-bearing on the POP device initially and weight-bearing will be gradually advanced over approximately the first 7-10 days as tolerated by the subject.  All standing and progressive static weight-bearing activities with the exoprosthetic components during this time will be directly supervised by the study physical therapist.  Once the subject is tolerating full weight-bearing in static standing, is cleared by the surgical team from a wound healing perspective, and cleared by physical therapy from a balance and safety perspective, dynamic weight-bearing in ambulation will begin.  All initial dynamic use of the exoprosthetic components and ambulation will be directly supervised by physical therapy.  The subject will be gradually progressed through their prosthetic training and each subject will be prescribed a minimum number of physical therapy treatment sessions following the Stage 2 procedure as outlined below.  The minimum treatment sessions for each enrolled subject will be:   1. Two sessions balance training: bilateral stance, weight shifts, single leg stance, perturbations, eyes open/eyes closed, small space movement 2. Two sessions gait training: smooth level surfaces, uneven surfaces, incline/decline, using upper extremities, etc. 3. Two sessions stair training: ascent/descent with and without handrails, step over step depending on prosthetic knee 4. Two sessions of return to activity: work, golf, low impact sports, etc. 5. One session home program: review   At the time of discharge the subject will undergo a physical examination including imaging, querying for adverse events, and clinical lab testing including serum drug screening, a physical therapy function evaluation, skin imaging and grading, and usage measures.  **e. Post Stage 2 Rehabilitation and follow-up**  Following discharge after Stage 2 Surgery, the subject will return to the George E. Wahlen Department of Veterans Affairs Medical Center at the following timepoints:   - 2 weeks ± 3 days - 6 weeks ± 7 days - 3 months  ± 14 days - 6 months ± 30 days - 12 months  ± 30 days - 24 months ± 200 Days - Long term follow-up arm - until next phase of study is approved.   At the Week 2 visits, subjects will undergo a physical examination including imaging, querying for adverse events, and clinical lab testing, a physical therapy evaluation, skin imaging and grading and skin swab and personal hygiene questionnaire.  Week 6 visits, subjects will undergo a physical examination including imaging, querying for adverse events, and clinical lab testing, a physical therapy evaluation, kinematic assessments, rehabilitation function, skin swab, personal hygiene questionnaire, psycho-social evaluation, skin imaging and grading, and usage measures.  At the Month 3, Month 6, 9 Month and Month 12 visits, subjects will undergo a physical examination including imaging, querying for adverse events, and serum pregnancy testing, mental health evaluation, social work evaluation, kinematic assessments, physical therapy evaluation, rehabilitation function testing, skin swab, personal hygiene questionnaire, skin imaging and grading, and usage measures.  At Month 24, subjects will undergo imaging (DEXA scan) and clinical lab testing.  At Long Term Follow-Up Arm, will be enrolled in this arm of the study to cover any visits needed for repair or maintenance of the device only. This will stay in effect until approval of the next stage of the study, estimated 24 months from August of 2019.  -After all study procedures are complete the participant will return home and will continue to be provided standard of care treatment for amputees through the VA Health Care System for their remaining lifetime.  During the informed consent process for our study, participants are also informed about our plans for tissue storage. Participants are informed that the principle investigator would like to save samples of their blood, skin, bone and skin swabs for future research regarding the POP implant they will receive as part of this study. Participants are informed that the collection of this tissue is required as part of the study. These samples will only be stored and used for research related to the POP implant and will not be banked for future unspecified research, but will be retained in order to answer upcoming research questions related to the POP implant.  They are informed that Dr. Sinclair and her study team will manage the tissue storage at the VA Salt Lake City Health Care System. Participants are also informed that their samples will be coded and identifiers stored in a separate place so that we can link the sample back to them if we need to.  We will inform participants that the sample may be shared with other researchers at the VASLCHCS and the University of Utah at the discretion of the investigator as part of the research performed related to the POP implant, but that they will not be identified by name. Contact information is listed if the participants have questions about the tissue storage at any time and they are also informed that no financial compensation will be available in the event the specimens are used to develop commercial products. Additionally, because research on this samples as it relates to the POP implant will not directly affect their health, the result of these tests will not be shared with participants.  Samples will be assigned a study specific identifier and lab number to be kept on the sample, with no PHI; these numbers can be linked to the patient’s research record only by IRB approved study team members (Drs. Sinclair and Stuart). No names, birthdates or any other patient identifiers are kept directly on the sample.  All patient information concerning the study and the tissue storage will remain confidential, kept in password protected computers and locked cabinets. Incoming research staff will be trained by the PI or research staff on the management of the de-identified samples in the tissue storage facility.  All records are kept in password protected computers and locked cabinets. Stored samples will be kept in a locked -80 freezer at the VASLCHCS. Only the PI and the research staff working with the study will have access to the samples.  There is a separate research study associated with this project that will be seeking consent and authorization to use these samples for a separate research project (Dr. Beck's micro biome study (IRB 73178).  This study will be required to obtain IRB approval prior to approaching participants about being in this optional microbiome study.  Investigators will be responsible for gaining IRB approval which includes any and all subsequent approvals required by the IRB including but not limited to data use agreements, data transfer agreements, etc. Specimens may only be used for future research that is approved by both parties.  In another study is created that would like to use these tissues, separate consent and authorization will need to be obtained.  The terms for, management, transfer and tracking of the specimens will be determined by Dr. Kubiak and the receiving PI before specimens are transferred in the event that consent and authorization is obtained from participants.  Transfer or destruction of specimens will be determined and managed by Dr. Sinclair if the need arises. In the event that Dr. Sinclair should leave the institution, all responsibility for the use and management of the specimens will fall to the ACOS of research Dr. Laurence Meyer and the Orthopaedic Department Chair, Dr. Charles Saltzman.  Dr. Sinclair will identify a non-conflicted peer subject to the approval of the Individual Conflict of Interest Committee to review the research and results prior to publication and to review this research and results at least annually and submit reports to the Conflict of Interest Office for review. \| \| --- \|   **Procedures performed for research purposes only:**   \|  \| \| --- \| |

**Statistical Methods, Data Analysis and Interpretation**

|  |
| --- |
| Up to 10 subjects will be enrolled in the study. This limited sample size is based on FDA guidance for early feasibility studies in the Early Feasibility Study investigational device exemption (IDE) pilot program. The statistical analysis of data will be limited to descriptive statistics for efficacy and safety parameters. Only intra-subject variability will be measured; there will be no inter-subject comparisons in this study. |
